# Molecular insights into triazole-based compounds for triple-negative breast cancer treatment

**DOI:** 10.1080/14756366.2026.2732821

**Published:** 2026-09-28

**Authors:** Ahamed Vilangalil, Sindhu Priya E. S., Sarad Pawar Naik Bukke, Sandhya V., Nafeesa Meha, Chandrashekar Thalluri, Kasolo Daniel, Allan Amooti Ahikiriza

**Affiliations:** a Department of Pharmaceutical Chemistry, Yenepoya Pharmacy College & Research Center, Yenepoya (Deemed to be University), Mangalore, Karnataka, India; b Department of Pharmacology, Yenepoya Pharmacy College & Research Center, Yenepoya (Deemed to be University), Mangalore, Karnataka, India; c Faculty of Health Sciences, Department of Pharmacy, Victoria University, Kampala, Uganda; d Department of Pharmaceutics, Yenepoya Pharmacy College & Research Center, Yenepoya (Deemed to be University), Mangalore, Karnataka, India; e Faculty of Pharmaceutical Science, Assam Down Town University (AdtU), Guwahati, Assam, India

**Keywords:** Triple negative breast cancer, triazole derivatives, multiple biological targets, molecular docking, therapeutic resistance

## Abstract

Triple-negative breast cancer (TNBC) is an aggressive breast cancer subtype lacking oestrogen, progesterone, and HER2 receptors, thus limiting the effectiveness of targeted therapies. Triazole compounds have emerged as promising anticancer agents because of their structural versatility, chemical stability, and ability to interact with multiple biological targets. This review summarises the chemistry, biological significance, and therapeutic potential of triazole derivatives against TNBC. It highlights key anticancer mechanisms, including apoptosis induction, cell cycle arrest, inhibition of angiogenesis and metastasis, and modulation of major oncogenic signalling pathways. The review also compares synthetic and naturally derived triazole compounds, emphasising structure–activity relationships and evidence from preclinical studies. In addition, it discusses the contribution of computational approaches, including molecular docking, virtual screening, and predictive modelling, to accelerate triazole-based drug discovery. Overall, this review underscores the potential of triazole pharmacophores as effective therapeutic candidates for TNBC and provides directions for future research to overcome therapeutic resistance.

## GRAPHICAL ABSTRACT

**Figure:**
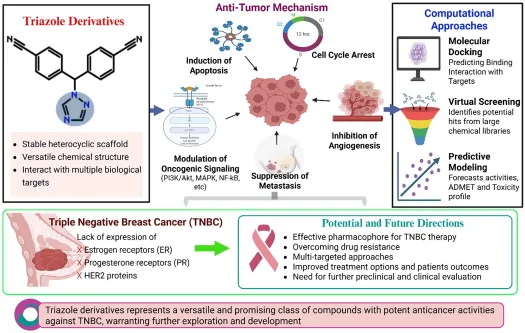

## Introduction

### Overview of triple-negative breast cancer (TNBC)

Triple negative breast cancer (TNBC) is a distinct and aggressive type of breast cancer in which there is no expression of oestrogen receptors (ER), progesterone receptors (PR), or human epidermal growth factor receptor 2 (HER2)^1^. This lack of hormone receptors reduces the efficacy of targeted hormonal therapy and HER2-directed therapy and contributes to the aggressive nature of triple-negative breast cancer. It accounts for about 10–15% of all breast cancer cases and is more common in younger women, especially those with mutations in the Breast Cancer Gene-1 (BRCA1). This disease is characterised by rapid growth of tumours, early metastasis, and a higher recurrence rate than other breast cancer subtypes^2^.

Triple-negative breast cancer has a high molecular diversity, which poses challenges to understanding its biology and developing effective therapies^2^^,^^3^. Unlike hormone receptor-positive breast cancer, triple-negative breast cancer does not have a single therapeutic target, which makes chemotherapy the current standard of care. Research has identified several triple-negative breast cancer subtypes, including basal, mesenchymal, and immunomodulatory subtypes, each with different molecular characteristics and therapeutic potentials (Table 1). Ongoing efforts to classify triple-negative breast cancers based on genomic and transcriptomic data are essential to identify new therapeutic objectives and treatment approaches for individual patients^4^.

**Table 1.** Molecular subtypes of triple-negative breast cancer (TNBC): pathophysiological.

TNBC subtype	Pathophysiological features	Key target proteins / pathways	Potential therapeutic agents	Representative cell lines	References
Basal-like 1 (BL1)	High proliferation; enriched in cell cycle and DNA damage response genes.	CDK1, BRCA1	PARP inhibitors (e.g. Olaparib, Talazoparib), CDK1 inhibitors	HCC1937, SUM149PT	7
Basal-like 2 (BL2)	Enriched in growth factor signalling and myoepithelial markers.	EGFR, MET	EGFR inhibitors (e.g. Erlotinib, Neratinib), MET inhibitors (Crizotinib)	HCC70, HCC1806	8
Mesenchymal (M)	Features related to EMT, cell motility, and differentiation	PDGFR, VEGFR, AXL, Src	Src inhibitors (Dasatinib), AXL inhibitors (Bemcentinib), VEGFR inhibitors (Pazopanib)	MDA-MB-231, BT-549	9
Mesenchymal Stem-like (MSL)	Similar to M subtype but with stemness and low proliferation gene expression	NOTCH1/2, JAK/STAT, Wnt/β-catenin	NOTCH inhibitors (Gamma-secretase inhibitors), Wnt inhibitors (LGK974), JAK inhibitors (Ruxolitinib)	SUM159PT, MDA-MB-436	10
Immunomodulatory (IM)	High immune cell signalling and cytokine expression	PD-L1, STAT1, JAK1	Immune checkpoint inhibitors (Atezolizumab, Pembrolizumab), JAK inhibitors	HCC38, BT-20	11
Luminal Androgen Receptor (LAR)	Driven by androgen receptor signalling; hormonal features despite TN status	AR, PI3K, mTOR	AR antagonists (Enzalutamide, Bicalutamide), PI3K inhibitors (Alpelisib), mTOR inhibitors (Everolimus)	MDA-MB-453, CAL-148	12
Unstable / Undefined	Genetically unstable; no dominant gene expression pattern	Variable; may include TP53, RB1 mutations	Chemotherapy, DNA-damaging agents (e.g. CHK1 inhibitors)	MDA-MB-468, HCC1143	13

**Table 2.** FDA-approved and clinically established therapeutic strategies relevant to TNBC.

Therapeutic agents/class	FDA-approved TNBC setting	Molecular target/mechanism	Major biological consequence	References
Pembrolizumab	High-risk early-stage TNBC with chemotherapy; unresectable locally recurrent/metastatic PD-L1-positive TNBC (CPS ≥10), with chemotherapy	PD-1 immune checkpoint inhibitor; blocks PD-1 interaction with PD-L1/PD-L2	Restores T-cell-mediated antitumor immune response	20
Sacituzumab govitecan-hziy	Metastatic TNBC; since June 2026 also first-line monotherapy in patients unsuitable for PD-1/PD-L1 therapy and first-line combination with pembrolizumab for PD-L1-positive TNBC	Trop-2-directed ADC delivering SN-38, a topoisomerase-I inhibitor	DNA damage, replication stress and tumour-cell apoptosis	21
Datopotamab deruxtecan-dlnk	Unresectable/metastatic TNBC in patients who are not candidates for PD-1/PD-L1 inhibitor therapy	Trop-2-directed ADC delivering a DXd topoisomerase-I inhibitor	Topoisomerase-I inhibition causes DNA damage, leading to tumour-cell death.	22
Olaparib	HER2-negative, germline BRCA-mutated high-risk early breast cancer in the adjuvant setting; also metastatic HER2-negative gBRCA-mutated breast cancer	PARP1/2 inhibition and PARP trapping	Impaired single-strand DNA repair and synthetic lethality in BRCA-deficient cells	23
Talazoparib	Locally advanced/metastatic HER2-negative breast cancer with germline BRCA mutation	Potent PARP inhibition/PARP trapping	Accumulation of DNA lesions and selective death of HR-deficient tumour cells	24
Carboplatin/ Cisplatin	Commonly used chemotherapy, particularly in selected early-stage/metastatic TNBC	DNA cross-linking/platinum-induced DNA damage	Replication blockage, DNA damage and apoptosis	25
Taxanes (Paclitaxel/ Docetaxel)	Chemotherapy backbone in TNBC	β-tubulin/microtubule stabilisation	Mitotic arrest and apoptosis	26
Anthracyclines (Doxorubicin/ Epirubicin)	Chemotherapy for early and advanced TNBC	Topoisomerase-II inhibition, DNA intercalation and free-radical-mediated damage	DNA double-strand breaks and apoptosis	27
Capecitabine	Selected patients with advanced/metastatic TNBC and residual disease after standard therapy	Prodrug converted to 5-fluorouracil, inhibiting thymidylate synthase and interfering with RNA/DNA synthesis	Antimetabolite-mediated inhibition of tumour proliferation	28
Letrozole/ Anastrozole	Hormone receptor-positive (ER-positive) breast cancer in postmenopausal women, including adjuvant treatment of early breast cancer and treatment of advanced/metastatic disease	Aromatase (CYP19A1) inhibition. The triazole nitrogen coordinates with the haem iron of CYP19A1, competitively inhibiting the conversion of androgens (androstenedione and testosterone) to oestrogens	Reduced oestrogen synthesis decreases oestrogen receptor activation, thereby inhibiting the proliferation of ER-positive breast cancer cells.	29

Triple-negative breast cancer is associated with poor prognosis and limited treatment options. Although progress in immunotherapy and targeted therapy has been made, the disease remains difficult to treat, particularly in cases of metastatic disease^5^. Despite initial response to chemotherapy, triple-negative breast cancer patients often relapse as drug resistance develops. Therefore, there is an urgent need to discover and develop new molecular therapies to improve the outcome of patients with this aggressive form of breast cancer^6^.

### Current treatment strategies and their limitations

The primary treatment approach for triple-negative breast cancer is usually a combination of surgery, chemotherapy, and radiotherapy. Neoadjuvant chemotherapy, given before surgery, is a common strategy for reducing the size of tumours and facilitating breast cancer surgery^14^. Then, adjuvant chemotherapy is often used to remove any remaining cancer cells. However, the lack of targeted treatment options for triple-negative breast cancer often leads to poor long-term results. While traditional chemotherapeutic agents such as anthracyclines and taxanes remain the backbone of TNBC therapy, they are often associated with serious systemic toxicity and have a poor survival profile in patients with metastatic disease. The intrinsic and acquired resistance to these drugs is a major problem, leading to recurrence and progression of cancer. The lack of predictive biomarkers also makes it difficult to select the most effective treatment regimen for each patient^15^.

In recent years, immune checkpoint inhibitors (ICI) targeting programmed death ligand 1 (PD-L1) have emerged as promising therapeutic modalities, especially in patients with high levels of expression of targeting programmed death ligand 1 (PD-L1). A combination of immune checkpoint inhibitors with chemotherapy has, in some cases, improved progression-free survival. However, not all patients with TNBC respond to immunotherapy, which highlights the need for additional biomarkers to predict response and to optimise treatment strategies^16^.

Poly(ADP-ribose) polymerase (PARP) inhibitors are an important class of targeted therapy for the treatment of breast cancer, particularly tumours harbouring germline BRCA1+ mutations or other homologous recombination (HR) defects. PARP inhibitors such as Olaparib and Talazoparib exploit faulty homologous recombination repair in breast cancer, BRCA1+ mutated, by inhibiting DNA repair mediated by PARP and trapping PARP in damaged DNA resulting in DNA accumulation, replication fork collapse, and synthetic lethality^17^.

The heterogeneity of TNBC continues to hamper efforts to develop drugs. Subtype-specific approaches are currently being explored, including targeted inhibitors of key signalling pathways such as PI3K, AKT, MTOR, and JAK-STAT^18^. These targeted therapies have the potential to improve clinical outcomes by providing a personalised approach to TNBC treatment. However, the lack of validated biomarkers and emergence of resistance mechanisms make it difficult to translate them clinically, which further highlights the urgent need for innovative therapeutic strategies^18^.

The therapeutic landscape for advanced TNBC has expanded substantially with the development of alternative therapy products. Sacituzumab govitecan is a trop-2 antibody consisting of a Trop-2-directed antibody that is associated with the SN-38 topoisomerase-I inhibitor. It delivers its cytotoxic payload primarily to tumour cells expressing Trop-2, followed by intracellular release of SN-38 and inhibition of topoisomerase I, resulting in DNA damage and death of tumour cells. Notably, in June 2026, the FDA expanded the role of TNBC by approving sacituzumab govitecan as first-line monotherapy in patients with unresectable locally advanced or metastatic TNBC and in combination with pembrolizumab in first-line PD-L1-positive disease (CPS >10). In addition, in May 2026, the FDA approved datopamab deruxtecan, another Trop-2-directed ADC, for the treatment of unresectable or metastatic TNBC in patients who are not candidates for PD-1 or PD-L1 inhibitors^19^.

PARP inhibitors are a molecularly targeted treatment strategy in patients with germline BRCA1+ mutations and HER2 negative breast cancer. Olaparib and Talazoparib inhibit the repair mediated by PARP and induce PARP binding, thereby increasing DNA damage. Tumour cells with defective homologous recombination repair, in particular those containing alterations of the BRCA1 or BRCA2 family, are particularly sensitive to this mechanism. Olaparib is approved by the FDA as adjunctive therapy in patients with deleterious or suspected deleterious BRCA-mutated HER2 negative germline breast cancer at high risk of developing early breast cancer after neoadjuvant chemotherapy. Talazoparib is also an approved PARP inhibitor in the treatment of HER2-negative, locally advanced or metastatic breast cancer in patients with germline BRCA mutations. Conventional chemotherapy, including taxanes, anthracyclines, platinum compounds, capecitabine, gemcitabine, and related agents, remains an important component of TNBC treatment, particularly for patients ineligible for biomarker-directed therapies^17^.

### The need for novel molecular therapeutics

Given the aggressive nature of triple-negative breast cancer and the limited treatment options, it is urgent to identify and develop new molecular therapeutics. Unlike hormone receptor-positive breast cancer, TNBC lacks targeted therapies, leading to chemotherapy-based treatment, which often leads to drug resistance and major side effects. Innovative therapeutic approaches are necessary to address these challenges by selectively targeting the biological pathways that are involved in the growth, metastasis, and resistance mechanisms of tumours^30^. Precision medicine strategies, including the identification of relevant genetic mutations and the development of targeted inhibitors, have the potential to transform the landscape of triple-negative breast cancer treatment^31^.

Emerging therapies such as small-molecule inhibitors, antibody conjugates, and immunotherapies are being investigated to provide safer and more effective alternatives^32^. In addition, rational drug design using computational methods has accelerated the discovery of new compounds with strong anticancer activity. Natural and synthetic small molecules, including triazole derivatives, have been shown to modulate key signalling pathways implicated in TNBC progression^33^. Combining these new therapies with existing treatments can enhance their effectiveness, reduce resistance, improve patient outcomes, and offer a more comprehensive approach to TNBC treatment. Given the aggressive nature of TNBC and the limited treatment options, it is urgent to identify and develop new molecular therapeutics^34^. Targeting key signalling pathways, inhibiting tumour proliferation, and inducing apoptosis are key strategies in the quest for more effective therapies. In this context, small-molecule inhibitors and bioactive compounds with selective cytotoxicity against TNBC cells have received considerable attention^35^.

### Triazole-based compounds in breast cancer therapy

Triazole derivatives, in particular 1,2,4-triazoles and 1,2,3-triazoles, have been identified as promising candidates for the development of anticancer medicines^36^. Their unique chemical structure provides a broad range of binding capacities, enabling effective interactions with diverse biological targets. Triazole compounds have shown significant anti-tumour activity by means of mechanisms including inhibition of enzymes, deoxyribonucleic acid (DNA) intercalation, and disruption of key cellular pathways^37^. Triazole derivatives have favourable pharmacokinetic properties, increased stability, and reduced toxicity, which contribute to their therapeutic potential. Recent research has highlighted the potential of triazole derivatives as selective inhibitors of triple-negative breast cancer proteins and offers a promising avenue for the development of targeted therapies for the treatment of breast cancer^38^.

One of the main mechanisms by which triazole derivatives exert an anticancer effect is through inhibition of enzymes critical to tumour cell proliferation. For example, certain triazole compounds target kinases and proteases, thereby disrupting the signalling pathways involved in the growth and metastasis of tumours^38^. In addition, triazoles have been shown to induce apoptosis by interfering with mitochondrial function and activating the caspase pathway. Such multi-targeted approaches increase their therapeutic effectiveness and reduce the likelihood of developing resistance to the product^39^.

The hydrophilic-lipophilic balance of triazole compounds can be fine-tuned to optimise their uptake and distribution within the cell. Structural changes, including the insertion of specific functional groups, were used to enhance the selectivity and efficacy of triazole derivatives against TNBC cells^40^. This adaptability makes them versatile candidates for combination therapies and may overcome resistance to conventional chemotherapy. Triazole derivatives have shown promising results in *in-vitro* and *in-vivo* against TNBC in recent studies. Molecular docking and in silico studies have identified triazole-based inhibitors targeting key proteins such as PARP, EGFR, and PI3K, which are often overexpressed in TNBC^41^. Triazole derivatives have synergistic effects in combination with immunocompetent inhibitors and chemotherapy, further enhancing their clinical potential^42^.

In view of the growing understanding of triazole chemistry and its use in cancer therapy, research continues to investigate new derivatives with greater specificity and reduced side effects^43^. The integration of computational drug design, bioassays, and structural-activity relationship (SAR) analysis will be key to the successful development of triazole-based TNBC therapeutics. By progressing through preclinical and clinical trials, these compounds offer a promising route to improving patient outcomes and represent a much-needed addition to the existing TNBC treatment landscape^44^ .Triazole derivatives, in particular 1,2,4-triazoles and 1,2,3-triazoles, have been identified as promising candidates for the development of anticancer medicines^45^. Their unique chemical structure provides a broad range of binding capacities, enabling effective interactions with diverse biological targets. Triazole compounds have shown significant anti-tumour activity by means of mechanisms including inhibition of enzymes, DNA intercalation, and disruption of key cellular pathways^46^. In addition, their favourable pharmacokinetic properties, increased stability, and reduced toxicity contribute to their therapeutic potential^47^. Recent research has highlighted the potential of triazole derivatives as selective inhibitors of TNBC proteins and offers a promising avenue for the development of targeted therapies for the treatment of tumours^48^.

Nonsteroidal aromatase inhibitors such as letrozole, anastrozole, and vorazole (Figure 1) are widely used as anti-cancer agents, especially in hormone-positive breast cancer. These compounds belong to the triazole group and act by reversibly binding to the aromatic enzyme CYP19A1, which inhibits the conversion of androgens (androstenedione and testosterone) to oestrogens. By significantly reducing circulating oestrogen levels, they reduce the growth and spread of tumours that depend on oestrogens. Letrozole and anastrozole are clinically approved third-generation inhibitors, and vorozole has demonstrated strong activity in preclinical and clinical studies. Their high selectivity, oral bioavailability, and favourable safety profile make them an essential component of endocrine therapy, particularly in postmenopausal women with breast cancer^49^.

**Figure 1.**
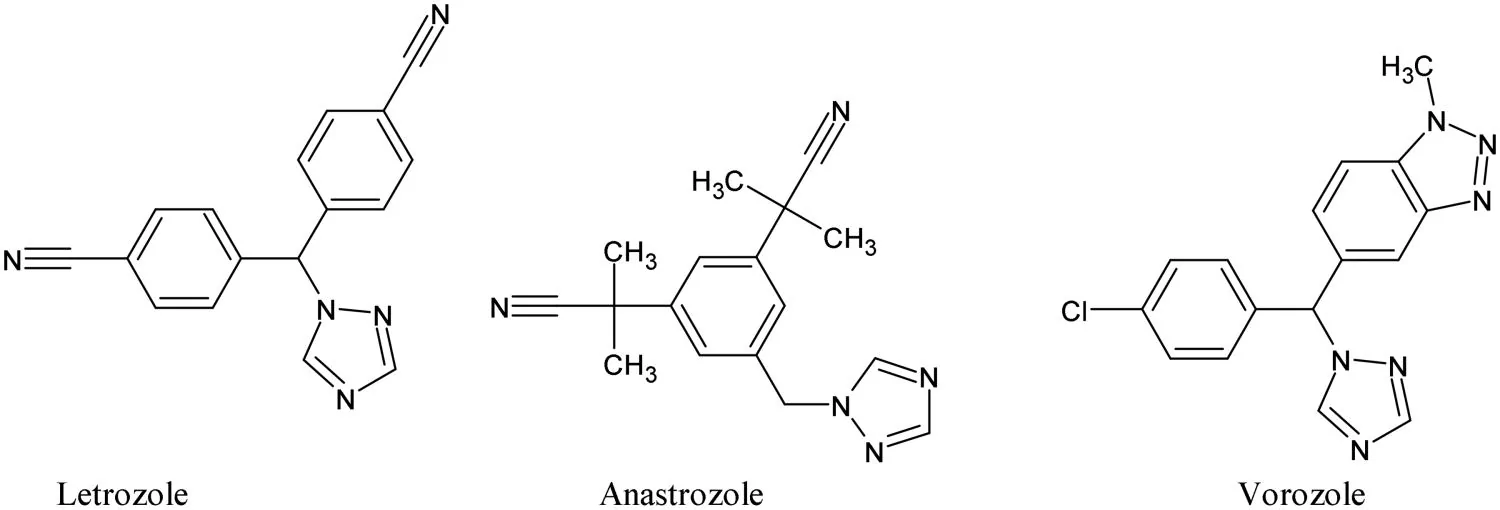
Structures of nonsteroidal aromatase inhibitors as anti-breast cancer agents: letrozole, anastrozole, and vorozole. Created using ACD/ChemSketch.

Triazole-containing medicinal products constitute an important class of clinically identified heterocyclic medicinal products, with 1,2,4-triazole being particularly relevant for antifungal, anticancer, and other medicinal product applications^50^. Several marketed medicinal products contain triazole pharmacophores, including Fluconazole, Itraconazole, Voriconazole, Posaconazole, Isoflavimazole, Letrozole, and Anastrozole^43^. The bioavailability of the triazole ring is due to its aromatic and electron-rich nature, the number of nitrogen atoms capable of hydrogen bonding, and its ability to engage in π-π interactions, hydrophobic interactions, and interactions with dipole groups with biological targets^51^. The incorporation of a triazole moiety may increase target affinity, metabolic stability, water solubility, and overall pharmacological activity, and additional lipophilic groups in itraconazole and Posaconazole increase hydrophobic interactions and broaden the spectrum of the antifungal agent^52^. Voriconazole maintains the basic interaction between triazoles and CYP51 but incorporates a fluoropyrimidine group, which contributes to its increased activity against several clinically important fungi^53^.

Triazole pharmacophores are also important in the development of anticancer drugs, particularly for inhibiting steroidogenic enzymes. Both Letrozole and Anastrozole, which are non-steroidal aromatase inhibitors, contain a 1,2,4-triazole ring and inhibit CYP19A1, an enzyme involved in the conversion of androgens to oestrogens. The nitrogen in triazole coordinates with the haem iron in aromatase, thereby inhibiting oestrogen biosynthesis and reducing the proliferation of breast cancer cells dependent on oestrogen^54^. Their SAR shows that the 1,2,4-triazole ring acts as a basic pharmacophore for metal binding. At the same time, the well-positioned nitrile or substituted aromatic groups enhance hydrophobic interactions at the active site of aromatase. The presence and location of aromatic rings are therefore important for maintaining potency, while substitution may affect lipophilicity, selectivity, and pharmacokinetic behaviour. The importance of the triazole nucleus in aromatase inhibitors has also spurred the development of numerous novel triazole derivatives targeting steroidal and cancer-related targets^55^.

Aromatase-inhibitory 1,2,4-triazole derivatives have been shown to inhibit a variety of cancer-related mechanisms, including inhibition of tyrosine kinases such as EGFR and VEGFR, modulation of PI3K and STAT signalling, inhibition of carbonic anhydrase and topoisomerases, inhibition of tubulin dynamics, apoptosis and autophagy, and interference with DNA-dependent processes^56^. Recent reviews suggest that structural optimisation of 1,2,4-triazoles usually involves N-substitution, incorporation of aryl or heteroaryl, modification of the linkers, fusion with other heterocycles, and molecular hybridisation^57^. Electron-withdrawing groups, such as halogens and nitriles, may enhance hydrophobic or electronic interactions, while electron-donating groups in appropriate positions may enhance hydrogen-bonding interactions and electronic complementarity. Hybridisation of the triazole ring with pharmacophores such as quinazoline, pyridine, indole, thiadiazole, and other heterocyclic systems frequently produces greater anticancer activity than the corresponding monocyclic structures^58^.

Traded triazole medicinal products provide valuable guidance and SAR principles for the rational design of new triazole derivatives for cancer. The central triazole ring provides a pharmacophore that is metabolically stable, polar, and aromatic, and rich in interactions. In contrast, modifications to peripheral aromatic groups, hydrophobic substituents, hydrogen-bond donors and acceptors, and molecular linkers may adjust potency, selectivity, and pharmacokinetic properties^59^. In particular, the metal-binding capacity of the triazole nitrogen makes the scaffold attractive to enzymes containing haem or metal centres, and its ability to engage in multiple noncovalent interactions facilitates the identification of targets for kinases and other tumour-associated enzymes. Recent literature confirms that 1,2,4-triazole derivatives are still under investigation against EGFR, VEGFR, aromatase, STAT, PI3K, and other molecular targets, supporting their potential as a versatile scaffold for the development of next-generation anti-cancer agents, including candidates for breast cancer and TNBC^60^.

Although several reviews and compendia have described the medicinal chemistry and pharmacological uses of triazole derivatives, this review is unique in that it specifically addresses breast cancer, in particular, triple-negative breast cancer. Most reviews have mainly provided a broad overview of 1,2,4-triazole-containing compounds in different therapeutic areas or discussed their general anticancer potential without systematically highlighting the molecular mechanisms specific to breast cancer. This review integrates the chemical structures, structural and activity relationships, molecular targets, mechanisms of action, and therapeutic relevance of triazole derivatives specifically for breast cancer. Particular emphasis is placed on clinically relevant targets and pathways associated with breast cancer, including aromatase (CYP19A1), EGFR, HER2, PI3K/Akt, PARP1, VEGFR, and apoptosis-related signalling pathways. The review further distinguishes itself by comparing marketed triazole-containing drugs with investigational triazole derivatives, thereby demonstrating how established pharmacophores can guide the design of novel anticancer candidates.

To the best of our knowledge, no previous review has comprehensively integrated breast cancer-specific SAR, molecular mechanisms, validated and emerging targets, marketed triazole-containing medicinal products, computational approaches, and recent experimental evidence on triazole derivatives, with particular relevance to TNBC. This review, aims to fill this gap by providing a targeted pharmaceutical chemist’s perspective on breast cancer treatments based on triazole.

## Triazole chemistry and its biological relevance

### Structural features of triazole compounds

Triazoles are five-membered heterocyclic compounds with two carbon atoms and three nitrogen atoms^71^. They are mainly found in two isomeric forms: 1,2,3-triazoles and 1,2,4-triazoles (Figure 2), each of which has a unique electronic structure and chemical reactivity^72^. These isomers are noted for their high nitrogen density, stability within aromatic structures, and ability to engage in hydrogen bonding and coordination, making them important pharmacophores in medicinal chemistry^73^. The difference between 1,2,3 and 1,2,4 triazoles provides a different electronic and physical arrangement of the ring nitrogens, and the ultimate anti-cancer activity is controlled by the interaction of this heterocyclic nucleus with the substituents and their respective pharmacodynamic structure^74^. The synthesis of 1,2,3-triazole derivatives has progressed considerably with the development of catalytic oxidation of click compounds, particularly the copper(I)-catalyzed azide cycloaddition (CuAAC)^75^. This reaction, independently discovered by Sharpless and Meldal in the early 2000s, allows the formation of 1,4-disubstituted triazoles^76^. The reaction is well tolerated under mild conditions, is tolerant of a wide range of functional groups, and affords high yields with minimal by-products, making it a preferred strategy for the synthesis of triazole-linked molecules in the fields of medicinal chemistry, materials science, and chemistry^77^.

**Figure 2.**
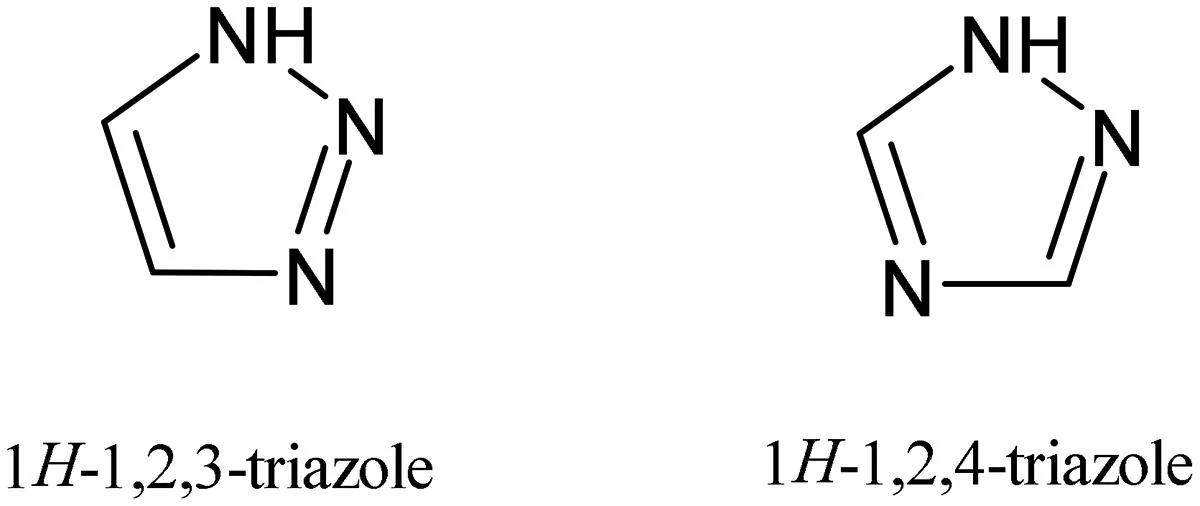
Structure of triazoles. Created using ACD/ChemSketch.

The synthesis of 1,2,4-triazole derivatives is predominantly achieved through cyclisation of hydrazide, thiosemicarbazide, amidrazone, or related nitrogen-rich precursors. A widely used synthetic sequence involves converting carboxylic acids or esters to the corresponding hydrazides with hydrazine hydrate, followed by treatment with carbon disulphide and a base to generate dithiocarbazate intermediates^78^. Subsequent cyclisation under acidic or oxidative conditions affords 1,2,4-triazole-3-thione derivatives, which can be further functionalised to generate diverse analogs. Alternatively, thiosemicarbazides can undergo cyclocondensation with carboxylic acids or their derivatives, while condensation of hydrazides with aldehydes followed by oxidative cyclisation provides another versatile route to substituted 1,2,4-triazoles. These methodologies permit modification at different positions on the heterocyclic ring and are therefore particularly valuable for structure–activity relationship studies^79^. Microwave-assisted cyclisation has further improved synthetic efficiency by reducing reaction times and, in several cases, increasing product yields. The choice of synthetic route depends on the desired substitution pattern, precursor availability, reaction conditions, and the physicochemical properties of the target triazole derivative^80^.

Modern synthetic strategies also investigate multi-component reactions (MCRs) and non-metallic protocols for triazole formation. MCR enables rapid assembly of triazole frameworks from simple precursors in one pot, thereby increasing efficiency and molecular complexity^81^. Transition metal-free conditions, such as thermal azide-alkyne cycloaddition or organocatalytic reactions, are becoming increasingly attractive for green chemistry applications. The wide range of synthetic methods, from classical to advanced, continues to expand the chemical space of triazole derivatives, making it easier to develop new drugs and materials^82^.

Structurally, the triazole rings serve as bioisosteres for amides, esters, and other heterocycles, enabling them to replace the labile groups of lead compounds without compromising their bioactivity. Their high electron density and ability to participate in π-π stacking and dipole interactions enable them to bind efficiently to a variety of biological targets, such as enzymes, receptors, and nucleic acids. In addition, triazoles resist hydrolytic and oxidative degradation, which improves the pharmacokinetic profile of triazole-containing medicinal products^83^.

### Mechanistic diversity of triazole derivatives

Mechanistically, triazole derivatives exhibit different modes of action depending on the mode of substitution and the nature of the scaffold^84^. They have been shown to inhibit enzymes such as cytochrome P450 enzymes, histone deacetylases (HdA), and carbonic anhydrase (CA)^85^. For example, the rings of 1,2,4 triazole in antifungal agents such as fluconazole and voriconazole bind to the iron haem in the 14-demethylase of the fungal enzyme lanosterol 14a, thereby interfering with the biosynthesis of ergosterol and cell membrane integrity^86^.

Triazoles also have a broad range of biological activities, including anticancer, antimicrobial, antiviral, anti-inflammatory, and anticonvulsant properties^87^. Their anti-tumour potential is particularly remarkable, as they may act as kinase inhibitors, DNA intercalators, and inducers of apoptosis^88^. The ability to fine-tune their electronic and steric properties by substitution makes them adaptable to a wide range of biological targets, increasing their selectivity and efficacy. In cancer, triazole-based inhibitors have shown promise against multiple molecular targets, including PI3K receptors, EGFR, and oestrogen receptors. These targets are involved in pathways regulating the cell cycle, proliferation, and angiogenesis. The modular nature of triazole synthesis allows for rapid optimisation of the lead compounds and facilitates structural relationship analysis studies, which are essential for the development of anticancer drugs^89^.

### Role of triazoles in drug development

In terms of drug development, triazoles make a significant contribution to the design of hybrid molecules and drug conjugates. Their chemical compatibility with a variety of binders and carriers makes them ideal for prodrug strategies, nanoparticle delivery systems, and antibody conjugates^60^. Moreover, the triazole-based binding is stable under physiological conditions, thereby reducing the risk of early drug release or degradation. Several FDA-approved medicines contain triazole rings, which highlights their therapeutic value. Remarkable examples include antifungal drugs (fluconazole, itraconazole), HIV medicines (rilpivirine), and cancer drug candidates under study. These successes highlight the versatility of triazoles not only in traditional small-molecule drugs but also in more complex therapeutic platforms, such as targeted therapies and immunomodulators^52^.

Recent studies have reported the formation of hybrid triazole-estradiol compounds that target the epidermal growth factor receptor (EGFR), a receptor often overexpressed in TNBC. The active substances are designed to inhibit EGFR signalling, which is associated with proliferation, migration, and poor prognosis in patients with TNBC^90^. Among these compounds, B (Figure 3) demonstrated potent cytotoxic activity against T-lymphocyte cell lines, including MDA-MB-231 and MDA-MB-468, with an IC_5_ in the low-micromolar range. These triazole derivatives not only block the activity of EGFR but can also modulate downstream pathways such as PI3K, AKT, and MAPK, thereby increasing their therapeutic potential^91^.

**Figure 3.**
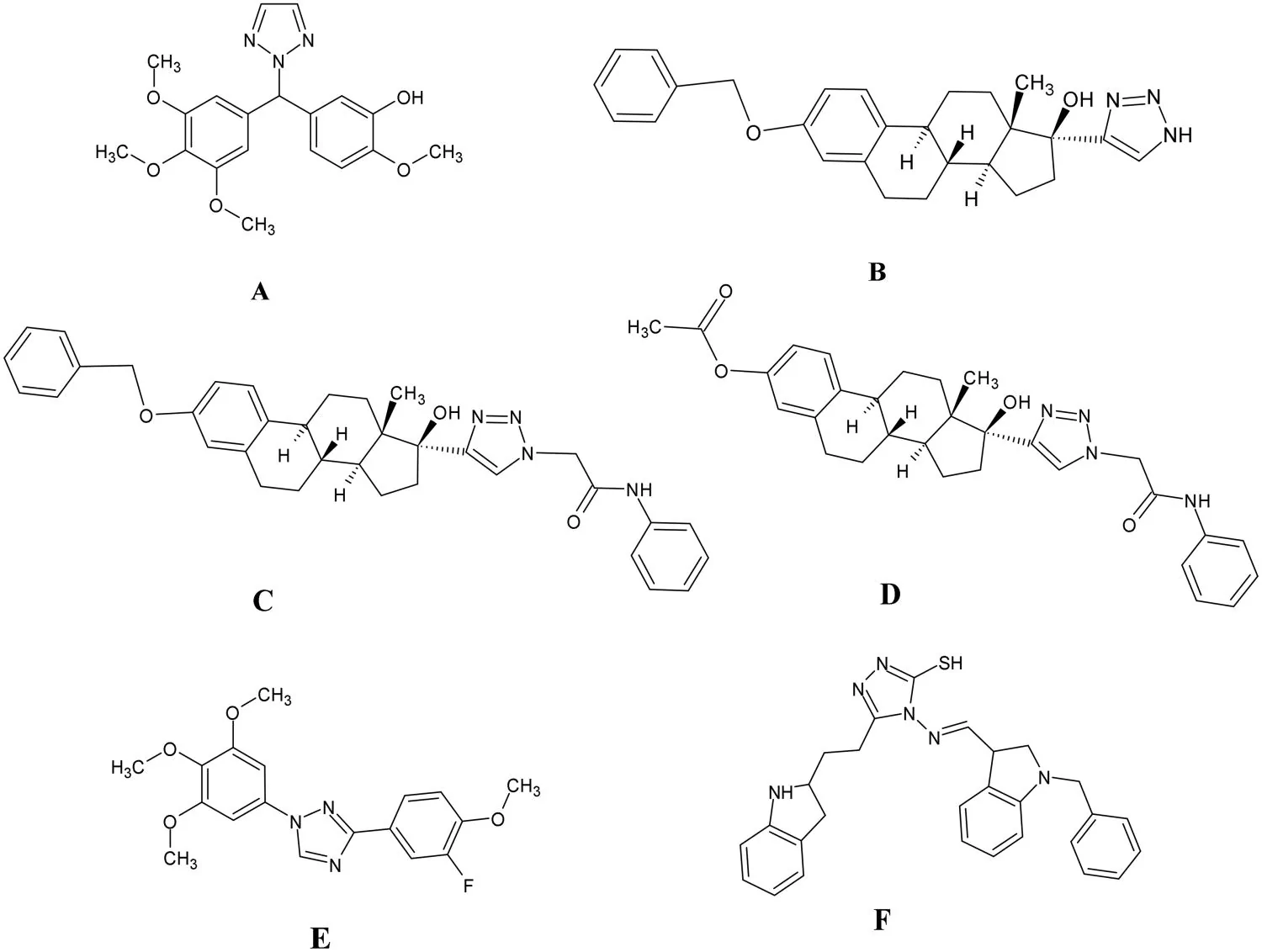
Structure of triazole derivatives. (A) 2-methoxy-5-[(2*H*-1,2,3-triazol-2-yl)(3,4,5-trimethoxyphenyl)methyl]phenol, (B) (13S)-3-(benzyloxy)-7,8,9,11,12,13,14,15, 16,17-decahydro-13-methyl-17-(1H-1,2,3-triazol-4-yl)-6H-cyclopenta[a]phenanthren-17-ol, (C) 2-(4-((13S)-3-(acetoxy)-7,8,9,11,12,13,14, 15,16,17-decahydro-17-hydroxy-13-methyl-6H-cyclopenta[a]phenanthren-17-yl)-1H-1,2,3-triazol-1-yl)-N-phenylacetamide, (D) (2-(4-((13S)-3-(benzyloxy)-7,8,9,11,12,13,14,15,16,17-decahydro-17-hydroxy-13-methyl-6H-cyclopenta[a] phenanthren-17-yl)-1H-1,2,3-triazol-1-yl)-N-phenylacetamide, (E) 3-(3-fluoro-4-methoxyphenyl)-1-(3,4,5-trimethoxyphenyl)-1*H*-1,2,4-triazole, (F) 5-(2-(1H-indol-3-yl)ethyl)-4-(((1-benzyl-1H-indol-3-yl)methylene)amino)-4H-1,2,4-triazole-3-thiol.

3–(34,5-trimethyloxyphenyl)-5-(N-methyl-3-indolel)-1,2,4-triazole (NMK-T-057) is a promising multi-drug candidate for the treatment of breast cancer, as it inhibits the signalling pathway of the cytochrome P450 system via the enzyme called notch. Its ability to inhibit proliferation, migration, EMT and stem formation while inducing apoptosis by autophagy, together with its potent anti-tumour activity in the murine 4T1 model, highlights its therapeutic potential to target the secretase-notch axis in TNBC. Importantly, its limited cytotoxicity to non-cancer cells further supports its potential for further preclinical development in the future^92^. The multifunctional potential of triazoles was also investigated in hybrid molecules that combine the triazole ring with other pharmacophores, such as histone deacetylase (HDAC) and PARP inhibitors, thereby increasing potency and tolerance to resistance. Taken together, these findings show that triazole-containing molecules represent a promising class of compounds for the targeted treatment of TNBC, and ongoing efforts are being made to improve their selectivity, efficacy, and drug-like properties^57^.

The development of triazole-containing medicinal products has been further supported by advances in computational modelling and molecular docking. These tools enable the prediction of binding affinity and the visualisation of triazole interactions with protein targets, thereby improving the efficacy of drug discovery. Machine learning approaches have also begun to predict biological activity from triazole substituents, thereby accelerating lead optimisation^93^. The triazole chemistry offers a solid framework for drug development, due to its synthetic availability, structural versatility, and broad range of biological activity. Its integration into modern medicinal chemistry continues to expand, spurred by innovations in synthetic methods and computational tools. Strategic inclusion of triazoles in therapeutic products is a powerful approach in the ongoing search for safer and more effective medicinal products^94^.

## Molecular mechanisms of triazole-based compounds in TNBC

### Targeting cell proliferation and growth pathways

Triazole compounds have been shown to interfere with the uncontrolled proliferation of TNBC cells by targeting growth-regulatory signalling pathways. One of the primary mechanisms is inhibition of tyrosine kinase receptors such as EGFR, which is commonly overexpressed in TNBC^95^. These receptors trigger a cascade that increases transcription of genes involved in cell division. By blocking the activation of receptors or downstream effects such as PI3K and mTOR, triazoles interfere with mitogenic signalling required for TNBC cell proliferation^96^. Triazoles have been shown to impair the activity of cyclin-dependent kinases (CDKs), particularly CDK4 and CDK6, which regulate G1 cell cycle progression. This inhibition reduces retinoblastoma (Rb) protein phosphorylation, thereby preventing the release of E2F transcription factors required for entry into S phase. As a consequence, TNBC cells are blocked in the G1 phase and are unable to proceed with DNA synthesis and replication^97^.

Triazole compounds frequently interfere with transcription factors, such as c-Myc, which regulate genes involved in cell metabolism and proliferation. Downregulation of c-Myc and suppression of the mTOR pathway not only reduces protein synthesis but also reduces the overall bioavailability of TNBC cells, thereby impairing their ability to grow and divide under the stress of the tumour microenvironment^98^.

### Apoptosis induction and cell cycle modulation

The triazole-based molecules induce apoptosis in TNBC cells primarily by an intrinsic mitochondrial pathway. This mechanism involves disruption of the potential of the mitochondrial membrane, resulting in cytochrome c release and activation of caspase-9, followed by caspase-3 and −7^99^. Triazoles modulate the balance of proteins in the Bcl-2 family by downregulating the anti-apoptotic (Bcl-2, Bcl-xl) and pro-apoptotic (Bax) proteins, and tipping the balance towards cell death^100^.

.Triazoles activate the extrinsic apoptotic pathway by activating death receptors such as FAS and TRAIL-R. Activation of these receptors initiates the caspase-8 cascade, which may either directly activate downstream caspases or potentiate the mitochondrial apoptotic pathway. This dual-pathway activation enhances the efficacy of triazole-induced apoptosis, especially in aggressive TNBC subtypes resistant to monotherapy^101^. Another critical effect of triazole derivatives on TNBC is the modulation of the cell cycle. These compounds may induce arrest at various cell cycle checkpoints, including G0, G1, and G2. This is achieved by inhibiting cyclin expression (e.g. cyclin D1 and cyclin B1) and activating checkpoint kinases such as p21 and p27. Arresting the cell cycle halts TNBC proliferation and predisposes cells to apoptosis, particularly when combined with DNA-damaging or other cytotoxic agents^102^.

### Anti-angiogenic and anti-metastatic properties

Triazole compounds have been shown to inhibit angiogenesis, a key process in tumour survival and spread. These molecules inhibit the expression of vascular endothelial growth factor (VEGF), a key mediator of tumour blood vessel growth. Reduced levels of VEGF reduce endothelial cell recruitment and the formation of new blood vessels, effectively depriving the tumour of oxygen and nutrients that are necessary for it to continue to grow and metastasise^103^. In addition, triazoles target matrix metalloproteinases (MMPs), particularly MMP-2 and MMP-9, which degrade the extracellular matrix and facilitate TNBC cell invasion and metastasis. By inhibiting the expression of MMP and enzyme activity, triazole compounds reduce cell invasion via the basement membrane and reduce the TNBC cells’ ability to metastasise to distant organs such as the lungs and liver^104^.

The triazole derivatives also affect epithelial-mesenchymal transition (EMT), which is a key process in the metastatic cascade. These compounds upregulate epithelial markers, such as E-cadherin, and downregulate mesenchymal markers, such as vimentin and N-cadherin. This reversal of EMT prevents the TNBC cells from acquiring the characteristic migratory and invasive characteristics of the tumour, thereby preventing its spread from the primary site of tumour. These anti-metastatic effects significantly increase the therapeutic potential of triazoles in the treatment of aggressive and metastatic TNBC^105^.

1,2,3-Triazole derivatives are an important class of cancer-fighting triazole compounds, since the three adjacent nitrogen atoms provide a polarised aromatic scaffold for hydrogen bonding and dipole-mediated interactions with biological targets. Their activity may be significantly altered by the presence of aryl, hetero-aryl, steroidal or other pharmacophore groups around the ring of the triazole. For example, the estradiol-linked derivatives B, C and D (Figure 3) exhibited cytotoxic activity against MDA-MB-231 T-cells with IC_5_ values of 8.12 ± 0.85, 21.18 ± 0.23 and 10.86 ± 0.69 mg, respectively. These compounds also showed activity against MDA-MB-468, with IC_5_ of 25.43 ± 3.68 mg for B. Mechanistic studies have shown interference of EGFR-related signalling and of the downstream ERK and MTOR pathways, accompanied by cell cycle arrest and apoptosis induction. These findings demonstrate that incorporation of the 1,2,3-triazole ring into a steroidal framework can generate compounds capable of modulating growth-factor signalling relevant to TNBC^90^.

The anticancer potential of 1,2,3 triazoles is highly dependent on the nature and the position of the substituents attached to the heterocyclic nucleus of the molecule. 5-((2*H*-1,2,3-triazol-1-yl)(3,4,5-trimethoxyphenyl)methyl)-2-methoxyphenol (A) reported to have an IC_5_ of approximately 0.074 micrograms against MDA-MB-231 cells, demonstrating the considerable potency achievable through appropriate pharmacodynamic optimisation (Figure 3 and Table 3)^106^. Compared to less active analogs, incorporating suitable aromatic and hydrophobic substituents may increase molecular complementarity with the target binding site, and adding hydrogen-bonding groups may strengthen interactions between the ligands and the protein. Similarly, the estradiol-triazole derivatives B and D were more active against MDA-MB-231 than C, indicating that substitutions, hydrophobicity, conformational orientation, and electronic properties of the active substance influence cellular potency. Thus, the 1,2,3-triazole ring should be considered a versatile pharmacophoric and linker unit rather than an independent determinant of anticancer activity^90^.

**Table 3.** Structural activity relationship (SAR) and mechanistic overview of triazole-based and related compounds in breast cancer therapy.

Drug / compound	Mechanism of action	Target	SAR	References
Letrozole	Aromatase inhibitor (non-steroidal)	Aromatase (CYP19A1)	The triazole nitrogen coordinates with the haem iron of CYP19A1, while the nitrile-containing aromatic groups occupy the hydrophobic binding region; appropriate spacing between the triazole and aromatic rings is essential for potency	60
Vorozole	Aromatase inhibition	Aromatase (CYP19A1)	The triazole nitrogen provides haem-iron coordination, whereas the nitrile and aromatic groups contribute hydrophobic interactions; electron-withdrawing substituents support favourable binding	61
Anastrozole	Reversible aromatase inhibition	Aromatase	Substitution around the aromatic region modulates hydrophobic interactions and orientation within the aromatase active site; appropriate steric bulk favours target binding	62
Fluconazole	Ergosterol synthesis inhibitor (off-target anticancer effects)	CYP enzymes, potential inhibition of breast cancer cell growth	The triazole nitrogens provide strong CYP haem coordination, while fluorophenyl groups increase lipophilicity and influence metabolic stability; structural modifications alter CYP affinity and selectivity	63
New Triazole-Derived HDAC Inhibitors	Induce apoptosis via histone deacetylase inhibition	HDAC1, HDAC6	The hydroxamate provides Zn²⁺ coordination, while the triazole and substituted aromatic group occupy the channel/cap region; increased hydrophobic interactions can enhance HDAC inhibition	64
1,2,3-Triazole-linked Combretastatin analogs	Microtubule disruption	β-tubulin	The triazole maintains an appropriate spatial relationship between the two aromatic rings while improving chemical stability; substitution on the aromatic rings strongly influences tubulin binding and cytotoxicity	65
1,2,3-Triazole-Tamoxifen Hybrids	ER modulation and antiproliferative activity	Oestrogen Receptor α (ERα)	Triazole incorporation can improve molecular rigidity and receptor interactions; linker length and aromatic substitution determine ER affinity and antiproliferative potency	66
Quinoline-triazole Hybrids	Topoisomerase inhibition and cytotoxicity	Topoisomerase II	Quinoline promotes π–π/DNA interactions, whereas the triazole contributes hydrogen-bonding interactions and controls linker geometry; halogen substitution can enhance lipophilicity and cytotoxic activity	67
Ruthenium-triazole complexes	Induce ROS and DNA damage; cytotoxicity.	DNA / Redox pathways	Modification of the triazole ligand and ancillary aromatic ligands changes metal-complex stability, cellular uptake, redox behaviour and cytotoxicity	68
Triazole-Quinazoline Derivatives	PI3K/AKT/mTOR inhibition	PI3Kα, mTOR, AKT1	Quinazoline provides the kinase-binding framework, while the triazole can act as a hydrogen-bonding linker; substitution around the quinazoline/triazole region modulates kinase affinity and cellular potency	69
Triazole-based PARP1 Inhibitors	Synthetic lethality in BRCA-deficient TNBC	PARP1	The triazole contributes hydrogen-bonding and polar interactions within the PARP catalytic pocket; increased rigidity and optimised hydrophobic substituents can improve PARP1 affinity and selectivity	70

**Table 4.** Triazole derivatives with anticancer activity against TNBC: cell lines, IC_50_ values, and molecular mechanisms.

Triazole derivatives	Triazole class/scaffold	TNBC cell line	IC₅。	Molecular target/mechanism	References
2-methoxy-5-[(2*H*-1,2,3-triazol-2-yl) (3,4,5-trimethoxyphenyl) methyl] phenol(A)	1,2,3-Triazole-Phenstatin hybrid	MDA-MB-231	0.074 μM	Antiproliferative/anti-mitotic activity	108
(13S)-3-(benzyloxy)-7,8,9,11,12,13,14,15, 16,17-decahydro-13-methyl-17-(1H-1,2,3-triazol-4-yl)-6H-cyclopenta[a]phenanthren-17-ol (B)	Estradiol–1,2,3-triazole	MDA-MB-231	8.12 ± 0.85 μM	EGFR; ERK/mTOR; apoptosis	90
(2-(4-((13S)-3-(benzyloxy)-7,8,9,11,12,13, 14,15,16,17-decahydro-17-hydroxy-13-methyl-6H-cyclopenta[a] phenanthren-17-yl)-1H-1,2,3-triazol-1-yl)-N-phenylacetamide (C)	Estradiol–1,2,3-triazole	MDA-MB-231	10.86 ± 0.69 μM	EGFR/ERK/mTOR; apoptosis	90
2-(4-((13S)-3-(acetoxy)-7,8,9,11,12,13,14, 15,16,17-decahydro-17-hydroxy-13-methyl-6H-cyclopenta[a]phenanthren-17-yl)-1H-1,2,3-triazol-1-yl)-N-phenylacetamide (D)	Estradiol–1,2,3-triazole	MDA-MB-231	21.18 ± 0.23 μM	EGFR/downstream signalling	90
3-(3-fluoro-4-methoxyphenyl)-1-(3,4,5-trimethoxyphenyl)-1*H*-1,2,4-triazole (E)	Diarylated 1,2,4-triazole	MDA-MB-231	17 ± 1.12 μM	BAX/mitochondrial apoptosis	107
5-(2-(1H-indol-3-yl)ethyl)-4-(((1-benzyl-1H-indol-3-yl)methylene)amino)-4H-1,2,4-triazole-3-thiol (F)	Indole–1,2,4-triazole	MDA-MB-231	48.22 μg/mL	FAK/PTK2 inhibition; apoptosis; G₁ arrest	108

In contrast, 1,2,4 triazoles contain nitrogen atoms at 1, 2 and 4 positions, resulting in a different electronic distribution and hydrogen bonding geometry than the isomer 1,2,3 triazole. This structural difference may significantly impact target recognition and biological activity. Diatabolite derivatives of 1,2,4-triazole compounds E (Figure 3) showing IC_5_ values of 17 micrograms against MDA-MB-231 cells. Structural modifications of aryl substituents have affected the antiproliferative response, with a clear correlation between substitution patterns and activity^107^. Other 1,2,4-triazole compounds have been designed to interact with cancer enzymes and signalling proteins, including EGFR, HER2, PI3K, PARP1, and aromatase. The 1,2,4-triazole scaffold provides a useful platform for developing structurally diverse compounds directed towards molecular pathways involved in TNBC proliferation, survival, invasion, and apoptosis^107^.

Further structural diversification was achieved by combining 1,2,4-triazole with other pharmacophores such as indole, thiadiazole, aryl, and Schiff base moieties. Such hybridisation may increase the molecular rigidity, increase the π-conjugation, introduce additional hydrogen bonding sites, and improve the interaction with the target protein’s hydrophobic regions. For example, indole-linked 1,2,4-triazole (F) showed an IC_5_ of approximately 48.22 mg/ml against MDA-MB-231 cells after 48 h of treatment (Figure 3)^108^. Mechanistic studies have linked its activity to inhibition of focal adhesion kinases (FAK and PTK2) accompanied by cell cycle arrest and apoptosis in the G_1_ phase. The compound also demonstrated anti-migratory effects, which are particularly relevant to TNBC due to its aggressive and metastatic phenotype. These observations show how combining a triazole nucleus with another bioactive heterocycle can alter bio-profiling and yield effects beyond simple cytotoxicity, including modulation of signalling, proliferation, migration, and apoptosis^109^.

Although a number of triazole derivatives have shown promising *in-vitro* activity against TNBC cell lines, relatively few compounds have been evaluated *in-vivo*. Therefore, available evidence should be differentiated at the experimental level, including computational modelling, *in vitro* cytotoxicity, mechanistic assays, and animal studies. For example, indole-1,2,4-triazole 4c has been further evaluated in preclinical studies, including *in vivo* toxicity and tolerability, which support its potential for further development. However, compounds such as A, B, and D (Figure 3) should be considered promising experimental candidates, particularly if their evidence is limited to cellular studies^90^. The comparison of 1,2,3 and 1,2,4 triazole derivatives suggests that the anti-tumour activity is determined not only by the triazole isomer but also by the complete molecular structure, including the heterocyclic nucleus, the substituents, the aromatic and hydrophobic groups, the molecular bonding, and other pharmacophores. Therefore, future developments should integrate structural and activity relationships, target-based studies, pharmacokinetic evaluation, toxicity assessment, and *in vivo* efficacy to identify triazole derivatives with real translational potential against TNBC.

### Interaction with key oncogenic pathways

#### PI3K/akt pathway

Triazole compounds exert anti-cancer effects in triple-negative breast cancer (TNBC) by targeting the phosphatidylinositol-3-kinase (PI3K) and protein kinase B (AKT) signalling pathways, which are often hyperactivated by PI3K mutations and by loss of the phosphatase and tensin homolog (PTEN). By inhibiting phosphatidylinositol-3-kinase (PI3K) or preventing the phosphorylation and activation of Protein Kinase B (AKT), triazole derivatives inhibit downstream signalling pathways, including the mechanistic target of rapamycin (mTOR), the transcription factor glycogen synthase-3 beta (GSK-3 beta), and the forkhead-box O (FOXO) transcription factors. This disruption reduces glucose metabolism, protein synthesis, and signalling pathways involved in cell survival, ultimately decreasing cell viability and increasing susceptibility to apoptotic stimuli in triple-negative breast cancer (TNBC)^110^.

#### NF-κB pathway

In triple-negative breast cancer (TNBC), triazole compounds also modulate the nuclear factor of kappa light chain (NF-κB) signalling pathway, which is critical for inflammation, cell survival, and resistance to apoptosis. These compounds inhibit the activity of IκB kinase (IKK), thereby preventing the phosphorylation and subsequent breakdown of the Kappa B-alpha inhibitor (IκB-alpha). As a result, nuclear factor of kappa light chain enhancer of B cells (NF-κB) remains in the cytoplasm, preventing its nuclear translocation and the transcription of survival and anti-apoptotic genes. Suppression of this pathway increases apoptotic signalling and contributes to decreased chemoresponsiveness in triple-negative breast cancer (TNBC)^111^.

#### MAPK pathway

In triple-negative breast cancer (TNBC), triazole compounds also affect the mitogen-activated protein kinase (MAPK) signalling pathway, which includes extracellular signal-regulated kinases 1 and 2 (ERK1 and 2), c-Jun N-terminal kinase (JNK), and p38 MAPK, and is a critical pathway for cell proliferation and apoptosis. These compounds inhibit mitogenic signalling by blocking phosphorylation of extracellular signal-regulated kinases 1 and 2 (ERK1 and ERK2), leading to reduced proliferation and cell-cycle arrest. At the same time, some triazole derivatives activate mitogen-activated protein kinase p38 (MAPK) or inhibit c-Jun N-terminal kinase (JNK) activity, thereby shifting signalling dynamics towards pro-apoptotic responses. By modulating multiple MAPK pathways in a coordinated fashion, triazoles disrupt the survival signalling networks and promote programmed cell death in triple-negative breast cancer (TNBC) (Figure 4)^112^.

**Figure 4.**
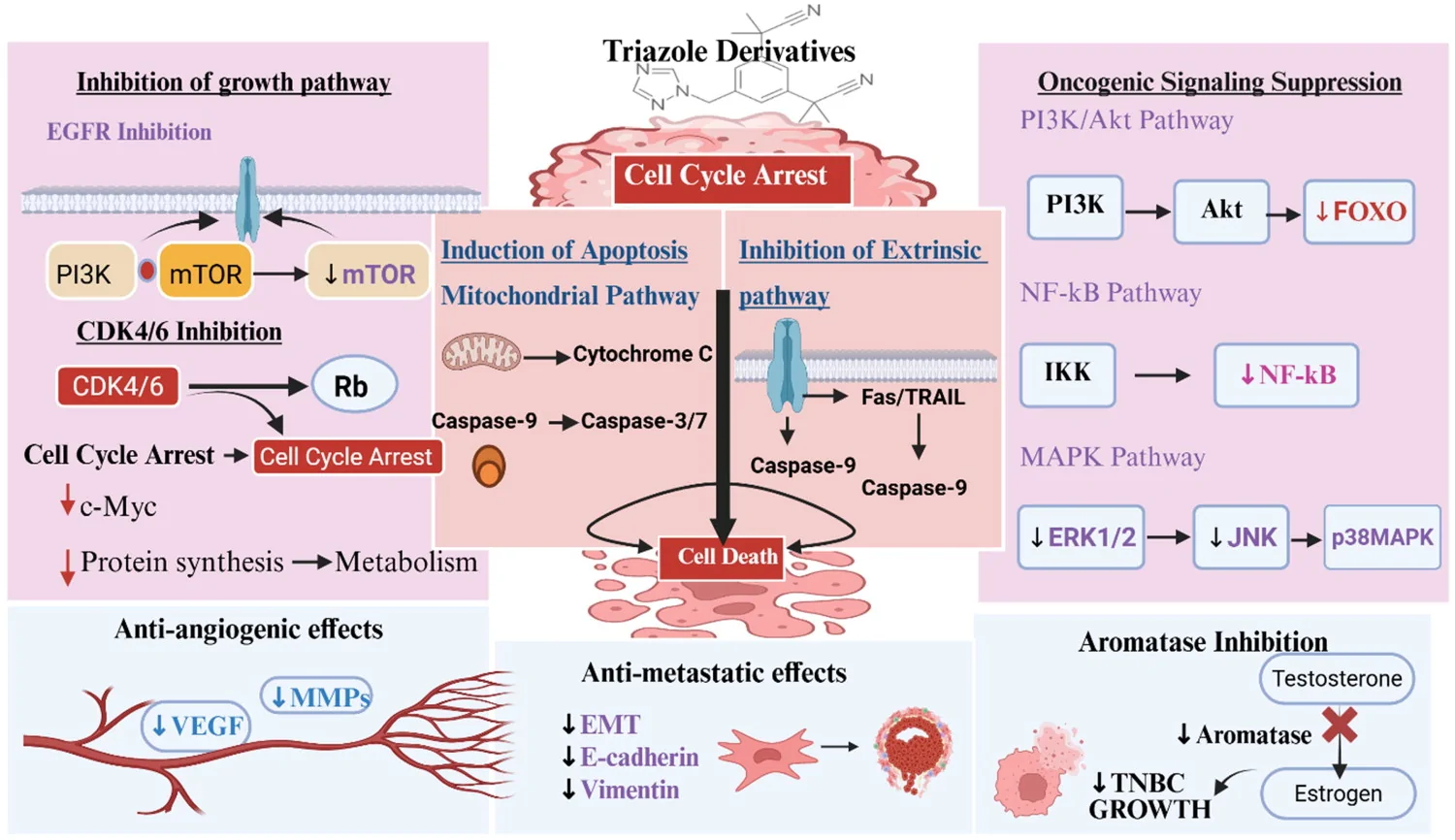
Mechanism of triazole derivatives in triple negative breast cancer (TNBC). Created with BioRender.com.

## Triazole derivatives as potential therapeutic agents for TNBC

### Synthetic and natural triazole-based molecules

Synthetic triazole derivatives have been extensively studied in cancer applications, including the treatment of triple-negative breast cancer (TNBC). Medicinal chemists have developed a number of scaffolds for 1,2,3 and 1,2,4 triazoles containing different aryl, hetero-, and alkyl substitutions that increase cytotoxicity against TNBC cells^113^. For example, compounds containing a fused triazole and thiadiazole backbone have a high selectivity against MDA-MB-231 cells due to mitochondrial dysfunction and inhibition of the polymerisation of microtubules^114^.

An important synthetic example is triazole conjugated chalcone, which combines two bioactive moieties. In TNBC lines, these hybrids demonstrated potent cytotoxicity by targeting tubulin polymerisation and inducing G2/M cell cycle arrest. The dual action of chalcone and triazole units leads to increased efficacy, demonstrating the potential of a rational hybrid design for the treatment of TNBC^115^. A second category includes quinoline analogs bound to triazole, which have been shown to reduce the viability of TNBC cells by inhibiting topoisomerase II and modulating DNA damage response proteins. These compounds often have excellent drug-like properties such as metabolic stability and favourable lipophilicity, which support their systemic use^116^.

Synthetic triazoles have been incorporated in multi-target cancer medicines in addition to monotherapy agents. HDAC inhibitors containing a triazole have been shown to reprogram epigenetic markers in TNBC cells, reversing silencing of tumour suppressor genes and increasing apoptotic signalling. These multi-functional formulations demonstrate the adaptability of triazoles as a key pharmacophore^117^. Natural triazole compounds, although less common, have been isolated from marine and fungal sources and exhibit mild antiproliferative activity. Triazolopyrimidines from marine mushrooms, for example, have shown promising anti-TNBC activity. Although the number of natural triazoles is limited, they have been an inspiration for the development of semi-synthetic analogs with increased potency and specificity^118^.

### Exploration of structure-activity relationships

SAR studies have played a key role in optimising triazole derivatives for the treatment of TNBC. These studies systematically modify the substituents on the triazole ring or on the adjacent linkages to determine their effects on bioactivity. For example, electron-withdrawing groups such as nitrosides and halogens in the para-position of the phenyl ring attached to triazole often increase cytotoxic potency by increasing cell permeability and potentiation of target binding^119^. Changes in the alkyl chain that connects the triazole ring to other pharmacophores also revealed significant trends in the SAR. Short, flexible linkers tend to favour improved cellular uptake and interaction with enzyme targets, while rigid or thickened chains may impair the enzyme’s bioavailability. In HDAC-triazole inhibitors, in TNBC models, the optimal length of the binding domain was critical to maintain zinc binding and maximise enzyme inhibition^120^.

SAR analysis of triazole-conjugated steroids showed that substitution patterns at the D-ring influence the selectivity for hormone-independent breast cancer cells. This is especially useful for TNBCs that lack hormone receptors. The addition of polarised side chains improved water solubility and reduced off-target toxicity, which was a common problem for early synthetic lead formulations^121^. Other areas of research in the field of SAR were heterocyclic systems fused or attached to a triazole nucleus. Triazole-benzimidazole hybrids and triazole-thiazole hybrids often show increased activity in the inhibition of DNA intercalation or kinase. These two pharmacologically active substances have synergistic effects that enhance cytotoxicity against aggressive TNBC cells while minimising the impact on normal mammary epithelial cells^122^.

.SAR data are now supported by *in situ* methods such as molecular docking and pharmacodynamic modelling. These approaches have sped up the identification of lead triazole compounds in TNBC that target PI3K, BRD4, or tubulin. Computational results help refine synthetic strategies, shorten synthesis time, and provide predictive models of compound efficacy across various preclinical TNBC models^123^.

### Preclinical evidence and experimental models

Triazole compounds have shown potent anti-neoplastic activity in various preclinical models. *In vitro* tests using TNBC cell lines, such as MDA-MB-231, BT-549, and HS5-1957, demonstrated that triazole derivatives can significantly reduce cell viability, induce apoptosis, and inhibit migration. These effects are often quantified by means of MTT, Annexin V, and PI stains or wound healing tests^113^.

Animal studies have further confirmed the anti-tumour efficacy of triazole compounds. For example, xenograft models implanted in immunodeficient mice with MDA-MB-231 cells showed reduced tumour volume when treated with triazole-based PI3K inhibitors. Pharmacokinetic evaluations in preclinical models were also performed to evaluate the bioavailability, half-life and tissue distribution of triazole compounds in mice, which demonstrated both efficacy and safety *in vivo*. Many derivatives exhibit favourable oral absorption and a moderate plasma half-life, making them suitable for possible oral use. Stability *in vivo* is often enhanced by structural modifications such as N-methylation and esterification^124^.

Histopathological examination of tumour tissues from treated animals often reveals increased apoptosis, decreased angiogenesis, and decreased proliferation markers such as Ki-67. These findings correlate with biochemical changes as detected by Western blotting, including decreased Akt and NF-κB signalling, which confirm the physiological activity of the compounds in a physiological setting^125^. In addition to xenografts, patient-derived tumour organoids and 3D spheroids are increasingly used to test the efficacy of triazoles. These advanced models provide a more realistic picture of tumour architecture and drug uptake profiles, and offer valuable translational insights. Several triazole candidates showed promising results in these models, which supported their progress to formal toxicity and IND-enabling studies^126^.

## Computational approaches in triazole drug discovery for TNBC

### Molecular docking, molecular dynamics, and virtual screening

Molecular docking has become an important structural approach for identifying and optimising triazole derivatives against molecular targets involved in triple-negative breast cancer (TNBC). Rather than being used only to rank compounds by docking score, docking may provide a structural explanation for why specific substituents or hybrid scaffolds enhance biological activity. EGFR is of particular relevance as it is often overexpressed in TNBC and is an important therapeutic target^127^. This approach provides structural information on ligand-target interactions and allows prioritisation of molecules with high binding values for synthesis and bioassays. For example, a study in 2023 used AutoDock Vina to assess a library of pyridines bound to triazole and to identify the top hits with nanomolar affinity to Akt1^128^.

The recent example is the design of the estradiol-1,2,3-triazole analogs Fz25, Fz57, and Fz200 as TNBC agents dependent on EGFR. Molecular docking against EGFR (PDB: 1M17) and molecular dynamics analysis supported a favourable interaction of the triazole scaffold in the EGFR binding region. Importantly, the calculations were supplemented with experimental data: Fz25 showed IC_50_ values of 8.12 ± 0.85 and 25.43 ± 3.68 mg against MDA-MB-231 and MDA-MB-468, respectively. These compounds inhibit EGFR and the downstream signalling pathways ERK and MTOR, and induce cell cycle arrest and apoptosis. Docking and molecular dynamics thus not only predicted binding but also helped promote the structure of estradiol-1,2,3-triazole as a new EGFR-directed scaffold for TNBC^90^.

.Recent advances in docking have included flexible modelling of ligands and receptors, thereby increasing the accuracy of predictions for dynamic targets commonly found in TNBCs. Software such as Schrödinger’s Glide and Gold now offers induced-fit protocols that capture more accurate conformational changes in target proteins upon ligand binding. This is particularly useful for triazole compounds, where the presence of multiple rotational bonds may affect binding orientation and efficacy^129^.

.In 2024, a hybrid 1,2,3-triazole[1,2,4]triazole[3,4-b][1,3,4]thiadiazine was developed via a chemical conjugation strategy and evaluated as an EGFR-targeted anticancer drug. The molecular docking and ADMET analyses were integrated, and compounds 7f and 7h demonstrated superior *in vitro* anticancer activity relative to erlotinib, along with potent EGFR inhibitory activity. The relevance of this strategy is that the computational analysis supported combining two heterocyclic pharmacophores rather than the traditional single triazole ring. Such a scaffold fusion may increase molecular rigidity, increase aromatic surface area, and create additional hydrogen-bonding and hydrophobic interactions, thereby providing new chemical structures for targeted cancer detection^130^.

Molecular docking may also be combined with experimental SAR to determine the structural parameters responsible for the enhanced TNBC activity. A recent series of indole-1,2,4-triazole hybrids is a good example. These compounds have been designed by binding to substituted 1,2,4-triazoles and have been tested against MDA-MB-231 cells and EGFR-TK (PDB: 1M17). Compound 4i, containing halogen substituents on indole and phenyl rings, showed the most potent antiproliferative activity, with an IC_50_ of 2,121 micrograms against MDA-MB-231. The docking results supported a favourable interaction with EGFR, and an ADMET analysis was conducted to evaluate the drug-likeness of the analogue. This example shows how computational analysis can guide the selection of electronically and hydrophobically optimised substituents, ultimately yielding a more effective chemical structure^107^.

Virtual screening of compound libraries has enabled the high-throughput identification of novel triazole compounds with potential anti-TNBC properties. Databases such as ZINC, PubChem, and CHEMBL were scanned in silico against targets relevant to TNBC. The 2024 screening campaign identified a number of kinase inhibitors containing triazole with selective activity against TNBC cell lines by binding to the ATP binding site of PAK1, a known regulator of TNBC invasion^131^.

For triazole derivatives, fragment-based virtual screening was also used. Researchers break down the lead triazole compounds into smaller pharmacophores, attach them individually, and then recombine the most promising fragments. This approach has resulted in the rational design of bidentate inhibitors that occupy two functional pockets on the protein surface simultaneously, an approach that increases both selectivity and efficacy for TNBC targets^132^. In addition, docking studies are now integrated with pharmacodynamic modelling to determine the structural parameters necessary for binding to TNBC targets. The pharmacodynamic model based on known EGFR triazole inhibitors in TNBC was helpful for the virtual screening and led to the identification of new scaffolds with favourable docking scores and *in vitro* validation. These integrative methods streamline the pipeline of discovery and reduce reliance on expensive experimental testing^133^.

### *In silico* ADMET profiling and optimization of drug-like properties

*In silico,* the ADMET (absorption, distribution, metabolism, excretion and toxicity) profile is essential to assess the drug-like properties of triazole derivatives in TNBC. Tools such as SwissADME, pkCSM and ADMETLAB 2.0 allow scientists to predict physicochemical properties, oral bioavailability, intracellular permeability and cytochrome P450 inhibition. This helps to exclude triazole compounds with adverse pharmacokinetics at an early stage of the discovery^134^. The series of Fz25, Fz57, Fz200 estradiol-1,2,3 triazole, where only Fz25 was reported to have a completely satisfactory *in silico* profile for ADMET.Fz25 was therefore the most promising compound for further computational and biological investigation, demonstrating how the ADMET filter can narrow the list to a more feasible candidate rather than selecting compounds solely for their cytotoxicity^90^.

.Recent studies have shown that many triazole derivatives meet the Lipinski Rule of Five criteria, indicating good oral bioavailability. However, some analogs exhibit high lipophilicity or poor solubility, which can be optimised by adjusting the polarity or introducing solubilising groups. A 2023 report optimised triazole-thiourea hybrids for TNBC by reducing logP values while maintaining target affinity, resulting in improved oral absorption predictions^135^. Predicting toxicity is another key element in in silico profiling. ADMET models now provide predictions for hepatotoxicity, cardiotoxicity (e.g. inhibition of the hERG channel), and genotoxicity. One study used ADMET Predictor to refine a number of triazole-related flavonoids, excluding those with high anticipated hepatic toxicity, thereby increasing the likelihood of clinical translatability. Triazole derivatives are frequently metabolised by CYP3A4, and computational tools can predict their metabolic sites and stability. This allows medicinal chemists to design analogs that are less susceptible to rapid degradation or formation of toxic metabolites^136^.

A recent series of indole-1,2,4-triazoles illustrates this approach, in which structural changes, including halogen substitutions, were evaluated experimentally for MDA-MB-231 activity and calculated for EGFR and ADMET binding. Compound 4q, which included halogen substitution in both aromatic regions, exhibited increased cellular activity, suggesting that computationally supported structural diversification may contribute to the identification of a more favourable lead architecture^107^. Plasma protein binding (PPB) and volume of distribution (Vd) predictions are useful for predicting *in vivo* pharmacokinetic behaviour. ADMET profiling has shown that some triazole derivatives exhibit high PPB, potentially decreasing the drug’s free concentration. Optimisation of the molecular weight and hydrophobic surface area has helped to improve these properties in recent triazole candidates for TNBC that have undergone preclinical evaluation^137^.

### QSAR, machine learning and AI-assisted triazole design

QSAR and machine learning approaches offer further opportunities to move from qualitative SAR observations to quantitative predictions of biological activity. Molecular descriptors such as molecular weight, logP, topological polar surface area, hydrogen bonding capacity, molecular volume and electronic parameters may be correlated to experimentally determined anticancer activity to determine structural properties related to efficacy. Although studies on AI targeting only triazoles against TNBC are still relatively limited, computational studies combining QSAR, docking, ADMET and molecular dynamics have demonstrated the potential of this integrated approach. For example, recent studies of drug discovery in breast cancer used QSAR models to identify physicochemical descriptors that affect activity, and then combined these predictions with docking and MD simulations to prioritise compounds. Such a workflow is particularly valuable for triazoles, as the substitution pattern can vary widely, creating large chemical spaces that are difficult to investigate experimentally^138^.

Recent work has used deep learning frameworks, such as graph convolutional networks (GCNs), to represent triazole molecules as graphs, enabling highly reliable bioactivity prediction against TNBC targets. For example, a study in 2024 used GCN to screen thousands of triazole compounds and to predict EGFR inhibitors, which were subsequently confirmed *in vitro* using TNBC cell lines^139^. AI and ML should be presented in the review as an emerging approach, rather than as evidence that large-scale trial candidates of triazole TNBC generated by AI have already been extensively tested.

Computer methods have contributed to the discovery of triazole drugs for TNBC in two main ways: optimisation of existing pharmacophores and generation of new chemical structures. Docking identifies target binding site regions that may contain substituents, MD simulations assess the stability of these interactions, and ADMET predictions exclude candidates with adverse drug-like properties. This information may then be transferred to structural modifications such as 1,2,3-triazole-estradiol hybrids, 1,2,4-triazole-indole hybrids, triazole-schiff base systems and fused structures of triazole-thiadiazole. The recent example of estradiol-1,2,3 triazole is particularly compelling as computational analyses supported EGFR binding and favourable ADM characteristics, and experimental studies subsequently demonstrated cytotoxicity and inhibition of EGFR and ERK and MTOR signalling in TNBC. Similarly, the development of hybrids of 1,2,3-triazole and triazolothiazide shows how computational scaffold fusion can generate structures with increased EGFR targeting potential^90^.

Reinforcement learning (RL) and generative adversarial networks (GANs) are used to create new triazole molecules with optimum properties. These models can learn from the desired outcomes (e.g. low toxicity, high target affinity) and propose improved candidates for further iteration. In addition, an AI-based study in late 2023 generated triazole hybrids with multiple target activities against both PI3K and mTOR, which were synthesised and shown to inhibit TNBC cell growth with minimal toxicity^140^. These platforms shall use ADMET predictive models, synthetic feasibility and target binding. Combining AI with cloud-based synthesis-planning tools enable the automated generation and evaluation of compounds, thereby shortening the discovery time for triazole-based TNBC therapies.

Explainable AI (XAI) tools such as SHAP (Shapley Additive Explanation) and LIME (local interpretable model-agnostic elucidation) are used to understand which triazole molecular structures are most responsible for activity or toxicity. This feedback loop helps to improve the design principles and to increase confidence in the compounds generated by the artificial arm for TNBC treatment^141^.

Computational approaches are useful for the design and optimisation of triazole derivatives, but docking scores and predicted ADMET profiles are not a direct determinant of therapeutic efficacy. The highly anticipated target binding may not translate into cellular activity due to poor permeability, efflux, metabolic instability, or toxicity. A series of erlotinib derivatives containing 1,2,3-triazole rings was designed using a structure-based method and combined with deep learning drug-target interaction models to predict their affinities. The derivatives were synthesised and tested for their IDO1 inhibitory activities. The results indicated that the activities of some compounds were better than those of erlotinib. Computational findings should be supported by synthesis, *in vitro* cytotoxicity, mechanistic studies, and *in vivo* evaluation. Machine learning and generative artificial intelligence can further optimise potency, selectivity, pharmacokinetic properties, and synthetic feasibility, but experimental validation is still necessary to confirm the anticancer potential of triazole derivatives developed computationally^142^.

## Triazole derivatives in combination therapy for TNBC

Triazole derivatives, once known for their excellent antimicrobial and antifungal properties, have now taken on a new role as effective anticancer agents due to their structural diversity and favourable pharmacokinetic profiles. These molecules have anti-tumour activity via multiple mechanisms, including inhibition of cell growth, induction of apoptosis, inhibition of angiogenesis, and regulation of intracellular signalling pathways^143^. In TNBC - an aggressive type of breast cancer without receptors for oestrogen, progesterone, or HER2 - the combination of triazole derivatives in combination therapies has been shown to be significant^144^. Chemoresistance, which is often seen in TNBC, remains a major barrier to clinical oncology. Conventional chemotherapeutic agents such as doxorubicin, paclitaxel, and cisplatin remain the mainstay of TNBC treatment, but their efficacy is often compromised by drug resistance and off-target toxicity. Triazole derivatives in combination with these anticancer medicines also showed synergistic anti-tumour activity, resulting in increased tumour cell killing and improved therapeutic efficacy^145^.

### Synergistic effects with chemotherapeutics and targeted therapies

Triazole derivatives have the potential to increase apoptosis induction by increasing the pro-apoptotic activity of chemotherapeutic agents by altering mitochondrial pathways, increasing the Bax-Bcl-2 ratio and activating caspases^146^. For example, some 1,2,3 triazole hybrids have been shown to sensitise TNBC cells to doxorubicin by increasing depolarisation of the mitochondrial membrane and increasing activation of caspase-3^147^. Increased uptake and intracellular retention of some triazole derivatives have been shown to involve interactions with cell membrane transport proteins or modulation of cell membrane permeability, thereby increasing intracellular concentrations of anticancer agents. This is especially important in TNBC cells, which frequently over-recycle efflux pumps such as P-glycoprotein (P-gp), reducing the bioavailability of the drug^148^. These derivatives can modulate DNA repair and ROS production, thereby interfering with tumour cell DNA repair mechanisms and making them more sensitive to DNA-damaging agents such as cisplatin. In addition, these compounds may increase reactive oxygen species (ROS) production, pushing cancer cells beyond their oxidative stress threshold and triggering apoptosis. In combination with conventional chemotherapy, triazole compounds have shown potential to interfere with the targeted therapy, particularly in drug-resistant TNBC models. For example, in BRCA-mutated TNBC, poly (ADP-ribose) polymerase inhibitors such as Olaparib are used, but resistance may arise from recombination recovery or increased drug efflux. Triazole-based compounds could target the compensatory mechanism or affect epigenetic modulators, thereby sensitising the tumour to PARP inhibition^149^.

Triazole derivatives have shown promise as co-treatments with epidermal growth factor receptor (EGFR) inhibitors, such as gefitinib. EGFR is highly overexpressed in TNBC and has a poor prognosis. Few triazole analogs exhibit inherent EGFR-inhibitory activity or can be hybridised with identified EGFR-targeting moieties, yielding dual-action compounds that inhibit proliferation and survival signalling pathways. These actions usually converge on cell-fate-determining nodes, such as the PI3K/Akt/mTOR and NF-κB pathways, which control survival, growth, and chemoresistance in TNBC. Compound Fz25, in particular, showed strong potency and favourable drug-like properties, supporting its further development for combination regimens^150^. Benzothiazole-triazole hybrids arrested the cell cycle and induced apoptosis in TNBC cells, primarily by inhibiting Bcl-2. This suggests they could develop chemotherapeutic agents that target apoptotic pathways more effectively^90^. Triazole-estradiol analogs have also been shown to kill TNBC cell lines by blocking EGFR and its downstream pathways. This suggests they could be more effective when combined with other EGFR-targeted therapies or chemotherapeutics^151^. Triazole-tethered boswellic acid derivatives exhibited potent anti-proliferative activity, with some compounds outperforming their parent molecules. These derivatives may act synergistically with other agents by targeting CHK1, a key regulator in cancer cell survival^152^.

Benzene sulphonamide- bearing bis-1,2,3-triazole derivatives 102 showed a significant cytotoxic effect, with an IC50 value of 1.81 μM, against breast cancer cell lines (MDA-MB231). 1,2,3-Triazoles containing sulphonamide moieties 103 and 104 showed significant anticancer activity against the MCF-7 cancer cell line, with IC50 values of 12.4 and 23.4 μM, respectively, compared to the standard drug doxorubicin (Figure 5)^153^.

**Figure 5.**
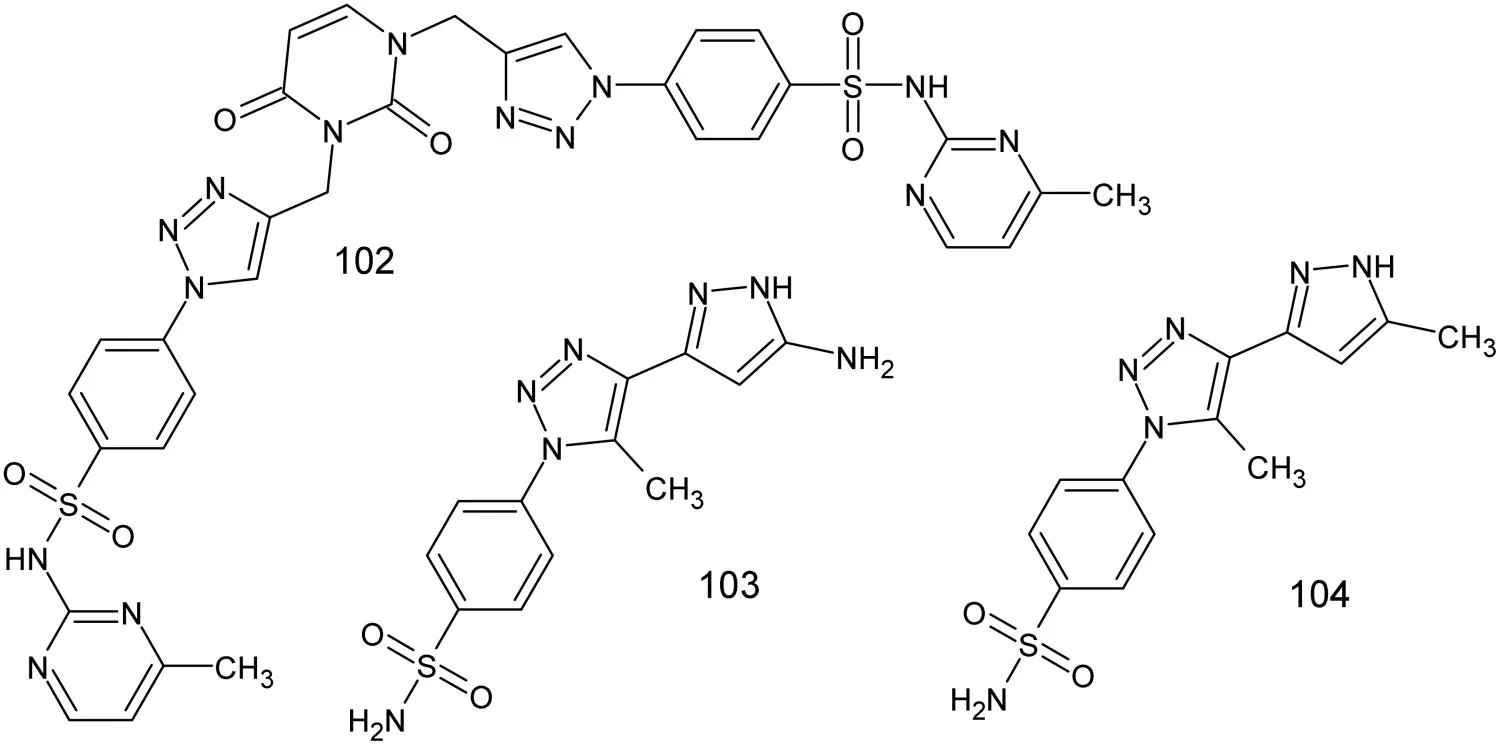
Benzene sulphonamide- bearing bis-1,2,3-triazole derivatives.

### Overcoming drug resistance in TNBC

The current problem in the treatment of TNBC is the onset of multidrug resistance (MDR), a process that seriously undermines the efficacy of chemotherapeutic and targeted drugs. In contrast to other subtypes of breast cancer, TNBC has no clear-cut hormonal or HER2 targets, leading to the use of non-specific cytotoxic agents. The rate of recurrence and lack of good long-term survival, though, are mainly a result of the rapid drug resistance acquired by the tumour^154^. The azole ring binds to transporter protein domains, preventing substrate binding or ATP hydrolysis. By inhibiting drug efflux, triazoles render resistant TNBC cells sensitive to standard chemotherapy and lower the required dose, reducing systemic toxicity. Cancer stem cells (CSCs), which can self-renew and have intrinsic resistance to treatment, also underlie TNBC recurrence and resistance^155^.

#### Blocking drug efflux pumps

Various triazole derivatives have been reported to inhibit P-glycoprotein, thereby restoring intracellular levels of chemotherapeutic agents such as doxorubicin or paclitaxel. The azole ring binds to transporter protein domains, preventing substrate binding or ATP hydrolysis. Through inhibiting drug efflux, triazoles make resistant TNBC cells sensitive to standard chemotherapy and lower the dosing required for therapy, reducing systemic toxicity^156^.

#### Targeting survival pathways and resistance-associated kinases

Some 1,2,3-triazole-linked hybrids, for instance, have been designed to inhibit EGFR and Akt, two kinases that are frequently hyperactive in TNBC. These dual inhibitors disrupt downstream survival signals and induce apoptosis, even in cells previously resistant to monotherapy. Triazole derivatives also regulate critical oncogenic pathways linked to drug resistance. Inhibition of NF-κB, a transcription factor implicated in inflammatory and anti-apoptotic gene regulation, has been found with some triazole analogs (Figure 6)^157^.

**Figure 6.**
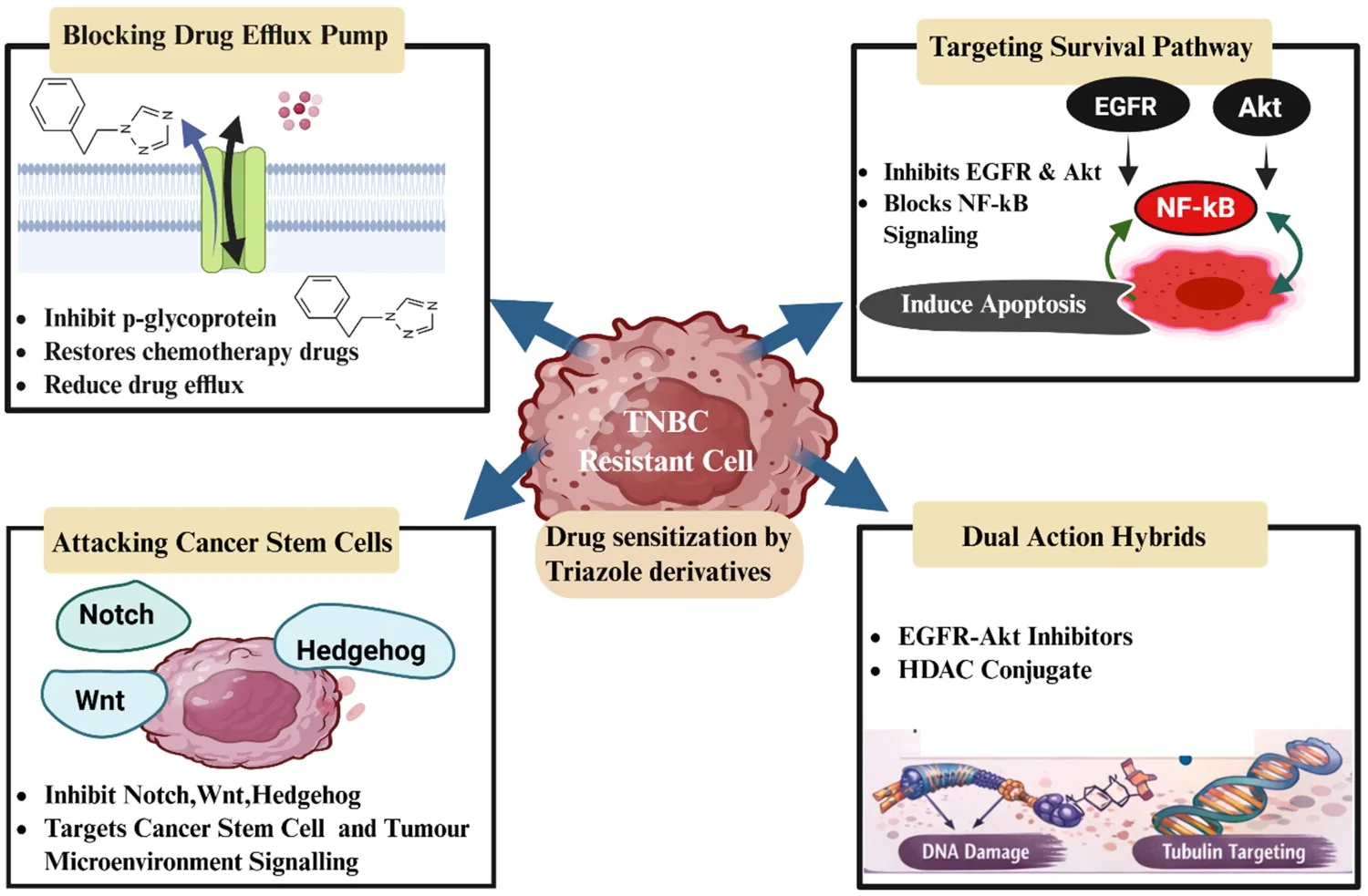
Overcoming drug resistance in TNBC with triazole derivatives. Created with BioRender.com.

#### Interfering with cancer stem cell (CSC) and tumor microenvironment signaling

Triazole-based agents can interfere with cancer stem cell (CSC) signalling pathways, including Notch, Wnt/β-catenin, and Hedgehog, that are involved in tumour relapse and drug resistance. Triazole derivatives may reduce the likelihood of resistance development by blocking several drug-resistance pathways, such as EGFR, Bcl-2, and CHK1, when used in combination with traditional treatments. Triazole scaffold-derived LPA1 antagonists inhibit TNBC cell invasion and migration but not cytotoxicity, offering a novel means to prevent metastasis and overcome drug resistance to conventional cytotoxic agents. Certain triazole derivatives are anti-inflammatory or anti-angiogenic, acting indirectly on TME-mediated resistance^158^.

#### Synthetic hybrids for dual action

Rational drug design has led to the synthesis of hybrid molecules containing triazole moieties tethered to known kinase inhibitors, histone deacetylase (HDAC) inhibitors, or tubulin-targeting compounds. The conjugates have additive or synergistic effects by targeting two resistance mechanisms simultaneously. Triazole-based dual EGFR-Akt inhibitors, for instance, have shown potential to overcome resistance in TNBC cells by inhibiting kinase phosphorylation and inducing DNA damage responses, in combination with DNA-damaging drugs. Triazole derivatives inhibit several pathways involved in drug resistance, including EGFR, Bcl-2, and CHK1, thereby reducing the likelihood of resistance development when administered alongside conventional therapies. LPA1 antagonists derived from triazole scaffolds suppress TNBC cell invasion and migration but not cytotoxicity, providing an innovative way to inhibit metastasis and overcome resistance to traditional cytotoxic drugs^159^.

### Potential for personalized medicine

TNBC is characterised by high molecular and phenotypic heterogeneity. This heterogeneity highlights the limitation of uniform treatment strategies and calls for personalised medicine. Precision or personalised medicine designs therapeutic interventions according to an individual’s characteristic genetic, molecular, and phenotypic tumour signature. In this shifting paradigm, derivatives based on triazole hold significant promise as chemotypes, with versatility and the potential to be structurally optimised to a particular patient’s needs^160^.

#### Chemical flexibility and modular design

One of the most essential features of triazole scaffolds is their chemical flexibility. The stable, bioactive 1,2,3-triazole ring, often formed via click chemistry, connects pharmacophores. This modularity makes it easy to quickly design a wide range of triazole-based molecules with different physical and chemical properties and biological activities^161^.

#### Target particular oncogenic mutations

Act selectively against overexpressed transporters or enzymes, including P-glycoprotein or cytochrome P450 isoforms. React to microenvironmental signals such as hypoxia, acidosis, or inflammation within the tumour microenvironment. Such structural versatility places triazole derivatives as prime candidates for designing tailored therapeutics in TNBC^136^.

#### *In silico* modeling and biomarker-based targeting

Recent computational drug design advances have enabled the identification and optimisation of triazole compounds based on individual patients’ biomarkers. Researchers can use molecular docking, QSAR modelling, and machine learning to predict how certain triazole derivatives will interact with target proteins in a patient’s tumour. This enables identification of the best compound or combination based on the patient’s proteomic and mutational profile. For example, docking simulations have shown that some triazole-spaced kinase inhibitors bind very well to mutant EGFR or Akt proteins, which are common in some TNBC subpopulations. In the same way, BRCA-mutated patients can be given PARP inhibitors as a top priority, making treatment even more personalised^162^.

#### Delivery systems for targeted nanocarriers

Encapsulating triazole agents in nanocarriers like liposomes, solid lipid nanoparticles, or polymeric micelles can make them more selective and less toxic to the whole body. The carriers can be modified with targeting ligands that specifically bind to tumour surface markers. These include Folate-conjugated systems for tumour cells overexpressing folate receptors.

CD44-targeted carriers to bind to cancer stem cells: Transferrin or integrin-based ligands for receptor-mediated uptake. The ligand-guided targeting strategy enables drug-carrying nanocarriers to target cancer cells while avoiding normal tissues, a fundamental principle of individualised medicine. Furthermore, the surface chemistry of the nanocarriers can be engineered to be responsive to environmental stimuli, such as pH or redox gradients, so that the drug is delivered only to the tumour site^163^.

#### Integration with omics data and clinical decision tools

Genomic, transcriptomic, and proteomic information obtained from patient biopsies can inform the rational selection of triazole compounds or delivery systems. For instance, tumours with hypoxic gene expression signatures might respond to triazole derivatives that release reactive oxygen species only when oxygen is scarce. AI-powered clinical decision support systems can match these molecular signatures to libraries of structurally diverse triazoles, speeding up the planning of precise treatment^164^.

## Challenges and future perspectives

### Pharmacokinetics and toxicological considerations

One of the main limitations of triazole derivatives in TNBC treatment is their unpredictable pharmacokinetics. Despite promising results *in vitro*, many candidates have poor systemic exposure due to insufficient solubility or rapid clearance. Challenges such as suboptimal gastrointestinal absorption, limited bioavailability, and high first-pass metabolism prevent them from advancing to clinical candidates. Addressing these issues requires formulation strategies such as nanoparticle encapsulation, prodrug design, or moiety incorporation to increase permeability^2^.

Toxicological liabilities remain a major concern during lead optimisation. Although triazoles are generally considered metabolically stable, some analogs may form reactive intermediates or accumulate in non-target tissues, leading to adverse effects. Studies have shown hepatotoxicity and hematological concerns with some triazole analogs, especially when administered at high doses. A comprehensive safety evaluation, including long-term toxicity and immunogenicity studies, is required before these agents enter clinical trials^165^.

The possibility of drug interactions complicates the use of the medicinal product, especially in patients receiving multiple therapies. Triazole compounds may inhibit or induce the activity of key cytochrome P450 enzymes, thereby affecting the metabolism of concomitantly administered medicinal products. The development of analogs with a reduced enzyme interaction profile or the use of combination therapy with careful monitoring may alleviate these problems, and early toxicogenomic profiling may help to predict and minimise unexpected adverse reactions^166^.

### Translational challenges from bench to bedside

A major translational barrier is the discrepancy between preclinical efficacy and clinical performance. Triazole compounds that show potent anti-TNBC activity *in vitro* often fail to replicate these results in animal models or human studies due to differences in the tumour microenvironment, the immune response, and the compounds’ pharmacodynamics. This divergence highlights the need for more predictive *in vivo* models, such as patient-derived xenografts (PDX) and humanised mouse models, to characterise the biology of human tumours better^167^.

.Regulatory barriers also play an important role in slowing the journey from bench to bedside. Novel triazole-based substances require extensive documentation, including synthesis, stability, pharmacology, and safety data. Due to the structural novelty of many triazole scaffolds, additional testing is often required, particularly in the first human studies. The lack of prior human use of many triazole derivatives may prolong the preclinical phase and delay entry into clinical use. Another key barrier is the design of clinical trials for TNBC, which is a highly heterogeneous and aggressive subtype of breast cancer. Patient stratification by molecular markers is critical but often underused in early-phase studies. Triazole compounds may only have an effect in specific subpopulations of TNBC, such as those with PI3K pathway activation or EGFR overexpression. The inclusion of companion diagnostics and biomarker-based selection criteria may significantly increase the likelihood of clinical success^168^.

### Future directions in triazole-based TNBC treatment

In the future, the therapeutic potential of triazole derivatives in TNBC may be enhanced by integrating multi-target strategies. Designing compounds that simultaneously inhibit kinases, epigenetic regulators, and immune checkpoints could yield synergistic effects. Researchers are increasingly investigating triazole hybrids that may modulate multiple pathways involved in TNBC progression, including immune evasion and DNA repair defects. This multi-target approach may overcome resistance mechanisms and increase response durability.

Emerging technologies such as artificial intelligence-guided drug design, 3D bioprinting of tumour models, and high-throughput organ-on-chip systems are poised to revolutionise the development of triazole drugs. These platforms allow rapid prototyping and real-time feedback on efficacy, toxicity, and pharmacokinetics. Combined with CRISPR-based genetic screening, these may help identify patient-specific deficits and match triazole scaffolds to different treatment regimens^169^.

## Conclusion

Triazole-based compounds have emerged as a promising class of anticancer agents for the investigation and potential treatment of triple-negative breast cancer (TNBC), owing to their structural versatility and diverse biological activities. Mechanistic studies suggest that selected triazole derivatives may modulate key oncogenetic signalling pathways, including PI3K, AKT, NF-κB, and MAPK, and may induce apoptosis and cell cycle arrest. In addition, several triazole compounds have been shown to have anti-angiogenic and anti-metastatic effects by inhibiting VEGFR and VEGFR-like signals, matrix metalloproteinases, and epithelial-mesenchymal transitions. These multi-target effects are particularly relevant in TNBC, which is characterised by significant molecular diversity, aggressive behaviour, drug resistance, and the limited availability of targeted therapies. Both synthetic and naturally inspired triazole derivatives have shown promising preclinical activity against TNBC cell models, but their potency, molecular targets, and mechanisms vary depending on the chemical scaffold and substitution pattern. Importantly, despite encouraging *in vitro* findings, the relatively limited *in vivo* validation of triazole compounds in TNBC models remains a major translational gap. Further structure–activity relationship studies, target validation, pharmacokinetic and toxicity investigations, and well-designed *in vivo* efficacy studies are therefore required to facilitate the development of triazole-based candidates into clinically relevant TNBC therapeutics.

Despite their biological promise, the translation of triazole derivatives from laboratory research into clinical use remains a challenge. Challenges include optimising pharmacokinetic properties, ensuring safety profiles, and resolving discrepancies between *in-vitro* and *in-vivo* efficacy. However, advances in computational modelling, including virtual screening, ADMET profiling, and the use of artificial intelligence, are helping to streamline drug discovery processes. SAR studies continue to inform rational modifications to increase selectivity and potency, and preclinical models, such as patient-derived organoids and xenografts, provide a more accurate platform for testing efficacy.

Integrating precision medicine approaches, multi-target design, and predictive biomarkers will be key to unlocking the full therapeutic potential of triazole compounds in TNBC. Cross-disciplinary cooperation and innovative technologies can help overcome translation hurdles and accelerate clinical development. As research continues to deepen our understanding of TNBC biology and triazole pharmacology, these compounds should contribute significantly to the development of new targeted therapies for this aggressive and currently underused subtype.

## Data Availability

Not applicable (this manuscript does not report data generation or analysis).

## Declaration

I hereby declare that this submission is entirely my own work, in my own words, and that all sources used in researching it are fully acknowledged and all quotations properly identified.

## References

[1] Ensenyat-Mendez M, Llinàs-Arias P, Orozco JIJ, Íñiguez-Muñoz S, Salomon MP, Sesé B, DiNome ML, Marzese DM. Current triple-negative breast cancer subtypes: dissecting the most aggressive form of breast cancer. Front Oncol. 2021;11:681476. 10.3389/fonc.2021.681476 PMC8242253 34221999

[2] Obidiro O, Battogtokh G, Akala EO. Triple negative breast cancer treatment options and limitations: future outlook. Pharmaceutics. 2023;15(7):1796. 10.3390/pharmaceutics15071796 PMC10384267 37513983

[3] Mahmoud R, Ordóñez-Morán P, Allegrucci C. Challenges for triple negative breast cancer treatment: defeating heterogeneity and cancer stemness. Cancers (Basel). 2022;14(17):4280. 10.3390/cancers14174280 PMC9454775 36077812

[4] Yam C, Mani SA, Moulder SL. Targeting the molecular subtypes of triple negative breast cancer: understanding the diversity to progress the field. Oncologist. 2017;22(9):1086–1093. 10.1634/theoncologist.2017-0095 PMC5599192 28559413

[5] Xiong N, Wu H, Yu Z. Advancements and challenges in triple-negative breast cancer: a comprehensive review of therapeutic and diagnostic strategies. Front Oncol. 2024;14:1405491. 10.3389/fonc.2024.1405491 PMC11165151 38863622

[6] Masci D, Naro C, Puxeddu M, Urbani A, Sette C, La Regina G, Silvestri R. Recent advances in drug discovery for triple-negative breast cancer treatment. Molecules. 2023;28(22):7513. 10.3390/molecules28227513 PMC10672974 38005235

[7] Lehmann BD, Bauer JA, Chen X, Sanders ME, Chakravarthy AB, Shyr Y, Pietenpol JA. Identification of human triple-negative breast cancer subtypes and preclinical models for selection of targeted therapies. J Clin Invest. 2011;121(7):2750–2767. 10.1172/JCI45014 PMC3127435 21633166

[8] Gaule P. c-Met/HGF in triple negative breast cancer [PhD Thesis]. [accessed 2026 Ma 11]. https://doras.dcu.ie/20582/.2015.

[9] Jaradat SK, Ayoub NM, Al Sharie AH, Aldaod JM. Targeting receptor tyrosine kinases as a novel strategy for the treatment of triple-negative breast cancer. Technol Cancer Res Treat. 2024;23:15330338241234780. 10.1177/15330338241234780 PMC10894558 38389413

[10] Gener P, Seras-Franzoso J, Callejo PG, Andrade F, Rafael D, Martínez F, Montero S, Arango D, Sayós J, Abasolo I, et al. Dynamism, sensitivity, and consequences of mesenchymal and stem-like phenotype of cancer cells. Stem Cells Int. 2018;2018:4516454. 10.1155/2018/4516454 PMC6199882 30405720

[11] Dewi C, Fristiohady A, Amalia R, Khairul Ikram NK, Ibrahim S, Muchtaridi M. Signaling pathways and natural compounds in triple-negative breast cancer cell line. Molecules. 2022;27(12):3661. 10.3390/molecules27123661 PMC9227697 35744786

[12] Zhao Y-X, Wang H, Zhang S-W, Zhang W-X, Jiang Y-Z, Shao Z-M. Enhancing therapeutic efficacy in luminal androgen receptor triple-negative breast cancer: exploring chidamide and enzalutamide as a promising combination strategy. Cancer Cell Int. 2024;24(1):131. 10.1186/s12935-024-03313-5 PMC11003165 38594722

[13] Shrestha M. Targeting vulnerabilities in RB-deficient breast cancer [PhD Thesis]. University of Toronto (Canada). [accessed 2026 Mar 11]. https://search.proquest.com/openview/55453b172e2d808c99cbf3283d39b580/1?pq-origsite=gscholar&cbl=18750&diss=y. 2023.

[14] Baskin AS, Huppert LA, Kelil T, Singer L, Mukhtar RA. The neoadjuvant approach to treatment of breast cancer: multidisciplinary management to improve outcomes. Surg Oncol Insight. 2024;1(2):100059. 10.1016/j.soi.2024.100059 PMC13215710 42211537

[15] Abuhadra N, Stecklein S, Sharma P, Moulder S. Early-stage triple-negative breast cancer: time to optimize personalized strategies. Oncologist. 2022;27(1):30–39. 10.1093/oncolo/oyab003 PMC8842325 35305094

[16] Wojtukiewicz MZ, Rek MM, Karpowicz K, Górska M, Polityńska B, Wojtukiewicz AM, Moniuszko M, Radziwon P, Tucker SC, Honn KV, et al. Inhibitors of immune checkpoints—PD-1, PD-L1, CTLA-4—new opportunities for cancer patients and a new challenge for internists and general practitioners. Cancer Metastasis Rev. 2021;40(3):949–982. 10.1007/s10555-021-09976-0 PMC8556173 34236546

[17] Zhou T, Zhang J. Therapeutic advances and application of PARP inhibitors in breast cancer. Transl Oncol. 2025;57:102410. 10.1016/j.tranon.2025.102410 PMC12142329 40359851

[18] Mallick S, Duttaroy AK, Dutta S. The PIK3CA gene and its pivotal role in tumor tropism of triple-negative breast cancer. Transl Oncol. 2024;50:102140. 10.1016/j.tranon.2024.102140 PMC11491976 39369580

[19] Tong Y, Fan X, Liu H, Liang T. Advances in Trop-2 targeted antibody-drug conjugates for breast cancer: mechanisms, clinical applications, and future directions. Front Immunol. 2024;15:1495675. 10.3389/fimmu.2024.1495675 PMC11563829 39555080

[20] Emens LA, Adams S, Cimino-Mathews A, Disis ML, Gatti-Mays ME, Ho AY, Kalinsky K, McArthur HL, Mittendorf EA, Nanda R, et al. Society for Immunotherapy of Cancer (SITC) clinical practice guideline on immunotherapy for the treatment of breast cancer. J Immunother Cancer. 2021;9(8):e002597. 10.1136/jitc-2021-002597 PMC8365813 34389617

[21] Wahby S, Fashoyin-Aje L, Osgood CL, Cheng J, Fiero MH, Zhang L, Tang S, Hamed SS, Song P, Charlab R, et al. FDA approval summary: accelerated approval of sacituzumab govitecan-hziy for third-line treatment of metastatic triple-negative breast cancer. Clin Cancer Res. 2021;27(7):1850–1854. 10.1158/1078-0432.CCR-20-3119 33168656

[22] Royce M, Shah M, Zhang L, Cheng J, Bonner MK, Pegues M, Miller CP, Leu L, Price LSL, Qiu J, et al. FDA approval summary: datopotamab deruxtecan-dlnk for treatment of patients with unresectable or metastatic, HR-positive, HER2-negative breast cancer. Clin Cancer Res. 2025;31(21):4405–4411. 10.1158/1078-0432.CCR-25-1388 PMC12393668 40864501

[23] Gourley C, Balmaña J, Ledermann JA, Serra V, Dent R, Loibl S, Pujade-Lauraine E, Boulton SJ. Moving from poly (ADP-Ribose) polymerase inhibition to targeting DNA repair and DNA damage response in cancer therapy. J Clin Oncol. 2019;37(25):2257–2269. 10.1200/JCO.18.02050 31050911

[24] Latif M, Mazhar S, Tasleem M, Alqurashi A, Arfan M, Ashraf Z, Zafar MS. Advances in PARP inhibition in improving outcomes of breast cancer, ovarian cancer, and other solid tumors: journey of discovery, development, and clinical updates of talazoparib. Drug Des Devel Ther. 2026;20:575152. volume 10.2147/DDDT.S575152 PMC13158130 42125218

[25] Petrović M, Todorović D. Biochemical and molecular mechanisms of action of cisplatin in cancer cells. Facta Univ Ser Med Biol. 2016;18(1):12–18.

[26] Aeluri M, Chamakuri S, Dasari B, Guduru SKR, Jimmidi R, Jogula S, Arya P. Small molecule modulators of protein–protein interactions: selected case studies. Chem Rev. 2014;114(9):4640–4694. 10.1021/cr4004049 24673632

[27] Richardson DS, Johnson SA. Anthracyclines in haematology: preclinical studies, toxicity and delivery systems. Blood Rev. 1997;11(4):201–223. 10.1016/s0268-960x(97)90020-5 9481450

[28] Page DB, Pucilowska J, Chun B, Kim I, Sanchez K, Moxon N, Mellinger S, Wu Y, Koguchi Y, Conrad V, et al. A phase Ib trial of pembrolizumab plus paclitaxel or flat-dose capecitabine in 1st/2nd line metastatic triple-negative breast cancer. NPJ Breast Cancer. 2023;9(1):53. 10.1038/s41523-023-00541-2 PMC10284878 37344474

[29] Rej RK, Roy J, Allu SR. Therapies for the treatment of advanced/metastatic estrogen receptor-positive breast cancer: current situation and future directions. Cancers (Basel). 2024;16(3):552. 10.3390/cancers16030552 PMC10854569 38339303

[30] Yin L, Duan J-J, Bian X-W, Yu S-C. Triple-negative breast cancer molecular subtyping and treatment progress. Breast Cancer Res. 2020;22(1):61. 10.1186/s13058-020-01296-5 PMC7285581 32517735

[31] Hossain F, Majumder S, David J, Miele L. Precision medicine and triple-negative breast cancer: current landscape and future directions. Cancers (Basel). 2021;13(15):3739. 10.3390/cancers13153739 PMC8345034 34359640

[32] Xu X, Zhang J, Wang T, Li J, Rong Y, Wang Y, Bai C, Yan Q, Ran X, Wang Y, et al. Emerging non-antibody–drug conjugates (non-ADCs) therapeutics of toxins for cancer treatment. Acta Pharm Sin B. 2024;14(4):1542–1559. 10.1016/j.apsb.2023.11.029 PMC10985036 38572098

[33] Aguirre-de Paz J, Lara-Almazán N, Ancira-Cortez A, et al. Potential anticancer activity of novel triazoles and related derivatives. Cham: Springer International Publishing; 2024.

[34] Samantaray A, Pradhan D. Novel combination therapy of osimertinib and Tupichinol E in triple-negative breast cancer: targeting EGFR and CDK4/6 pathways. Asp Mol Med. 2025;5:100069.

[35] Bahar ME, Kim HJ, Kim DR. Targeting the RAS/RAF/MAPK pathway for cancer therapy: from mechanism to clinical studies. Signal Transduct Target Ther. 2023;8(1):455. 10.1038/s41392-023-01705-z PMC10725898 38105263

[36] Guan Q, Gao Z, Chen Y, Guo C, Chen Y, Sun H. Structural modification strategies of triazoles in anticancer drug development. Eur J Med Chem. 2024;275:116578. 10.1016/j.ejmech.2024.116578 38889607

[37] Laamari Y, Bimoussa A, Chagaleti BK, Saravanan V, Alotaibi SH, Alotaibi FM, Mk K, Oubella A, Itto MYA, Auhmani A, et al. Thymol-1, 2, 3-triazole derivatives: network pharmacology, molecular simulations and synthesis targeting breast cancer. J Mol Struct. 2025;1321:140060.

[38] Chirra S, Gondru R, Manne M, Azam M, Al-Resayes SI, Manchal R, Narsimha S. Synthesis of [1, 2, 4] triazolo [3, 4-b][1, 3, 4] thiadiazine-1, 2, 3-triazoles as potent EGFR targeting anti-breast cancer agents. J Mol Struct. 2024;1306:137803.

[39] Othmène YB, Monceaux K, Belhadef A, et al. Triazole fungicide tebuconazole induces apoptosis through ROS-mediated endoplasmic reticulum stress pathway. Environ Toxicol Pharmacol. 2022;94:103919. 10.1016/j.etap.2022.103919 35753672

[40] Arvas B, Ucar B, Acar T, Varli HS, Arvas MB, Aydogan F, Yolacan C. Synthesis of novel coumarin-triazole hybrids and first evaluation of the 4-phenyl substituted hybrid loaded PLGA nanoparticles delivery system to the anticancer activity. Nanotechnology. 2024;35(30):305602. 10.1088/1361-6528/ad403e 38636487

[41] Youssef MF, Nafie MS, Salama EE, Boraei ATA, Gad EM. Synthesis of new bioactive Indolyl-1,2,4-triazole hybrids as dual inhibitors for EGFR/PARP-1 targeting breast and liver cancer cells. ACS Omega. 2022;7(49):45665–45677. 10.1021/acsomega.2c06531 PMC9753112 36530255

[42] Gao G, Li J, Cao Y, Li X, Qian Y, Wang X, Li M, Qiu Y, Wu T, Wang L, et al. Design, synthesis, and biological evaluation of novel 4, 4′-bipyridine derivatives acting as CDK9-Cyclin T1 protein-protein interaction inhibitors against triple-negative breast cancer. Eur J Med Chem. 2023;261:115858. 10.1016/j.ejmech.2023.115858 37837671

[43] Guan Q, Xing S, Wang L, Zhu J, Guo C, Xu C, Zhao Q, Wu Y, Chen Y, Sun H, et al. Triazoles in medicinal chemistry: physicochemical properties, bioisosterism, and application. J Med Chem. 2024;67(10):7788–7824. 10.1021/acs.jmedchem.4c00652 38699796

[44] Turky A, Sherbiny FF, Bayoumi AH, Ahmed HEA, Abulkhair HS. Novel 1,2,4-triazole derivatives: design, synthesis, anticancer evaluation, molecular docking, and pharmacokinetic profiling studies. Arch Pharm (Weinheim). 2020;353(12):e2000170. 10.1002/ardp.202000170 32893368

[45] Al Sheikh Ali A, Khan D, Naqvi A, Al-Blewi FF, Rezki N, Aouad MR, Hagar M. Design, synthesis, molecular modeling, anticancer studies, and density functional theory calculations of 4-(1,2,4-Triazol-3-ylsulfanylmethyl)-1,2,3-triazole derivatives. ACS Omega. 2021;6(1):301–316. 10.1021/acsomega.0c04595 PMC7807778 33458482

[46] Narasimhachar BC, Ravish A, Beeraka NM, Pookunoth BC, Basappa S, Vishwanath D, Rangappa KS, Nagaraja O, Madegowda M, Chandrashekara PG, et al. Development of novel pyrimidine-thio-triazoles targeting EGFR in breast Cancer cells via one-pot copper-catalyzed 1, 3-dipolar cycloaddition. Results Chem. 2025;14:102150.

[47] Yuriy K, Kusdemir G, Volodymyr P, Tüzün B, Taslimi P, Karatas OF, Anastasia K, Maryna P, Sayın K. A biochemistry-oriented drug design: synthesis, anticancer activity, enzymes inhibition, molecular docking studies of novel 1,2,4-triazole derivatives. J Biomol Struct Dyn. 2024;42(3):1220–1236. 10.1080/07391102.2023.2253906 37671856

[48] Wu X, Wang J, Xia S, Cheng S, Shi Y. 1,2,3-triazole derivatives with anti-breast cancer potential. Curr Top Med Chem. 2022;22(17):1406–1425. 10.2174/1568026622666220415225334 35473548

[49] Chumsri S, Howes T, Bao T, Sabnis G, Brodie A. Aromatase, aromatase inhibitors, and breast cancer. J Steroid Biochem Mol Biol. 2011;125(1-2):13–22. 10.1016/j.jsbmb.2011.02.001 PMC3104073 21335088

[50] Zhou C-H, Wang Y. Recent researches in triazole compounds as medicinal drugs. Curr Med Chem. 2012;19(2):239–280. 10.2174/092986712803414213 22320301

[51] Almohaywi B, Ahmed HEA, Kotb A, Ashraf A, Alsedawy M, Habib E-SE, Aljohani AKB, Alosaimi KA, Alharbi AS, Miski SF, et al. Triazole-functionalized compounds as promising antifungal agents: synthesis, biological evaluation, and mechanistic insights. Naunyn Schmiedebergs Arch Pharmacol. 2025;398(12):17573–17593. 10.1007/s00210-025-04350-1 40512216

[52] Peyton LR, Gallagher S, Hashemzadeh M. Triazole antifungals: a review. Drugs Today (Barc). 2015;51(12):705–718. 10.1358/dot.2015.51.12.2421058 26798851

[53] Choi JY, Podust LM, Roush WR. Drug strategies targeting CYP51 in neglected tropical diseases. Chem Rev. 2014;114(22):11242–11271. 10.1021/cr5003134 PMC4254036 25337991

[54] Ahmad I, Shagufta. Recent developments in steroidal and nonsteroidal aromatase inhibitors for the chemoprevention of estrogen-dependent breast cancer. Eur J Med Chem. 2015;102:375–386. 10.1016/j.ejmech.2015.08.010 26301554

[55] Hashmi SZ. Triazoles as enzyme inhibitors: synthetic advances, mechanism of action, molecular docking studies and structure-activity relationships. Mol Divers. 2026;4:1–46. 10.1007/s11030-026-11645-9 42400758

[56] Sharma M, Gandhi A, Sharma K. Triazole-based inhibitors for breast cancer: biological activity, SAR and synthetic strategies. Mol Divers. 2026;19:1–47. 10.1007/s11030-026-11583-6 42154401

[57] Rohilla S, Goyal G, Berwal P, Mathur N. A review on indole-triazole molecular hybrids as a leading edge in drug discovery: current landscape and future perspectives. Curr Top Med Chem. 2024;24(18):1557–1588. 10.2174/0115680266307132240509065351 38766822

[58] Mysłek M, Kaczmarek A, Świątek P, Czyżnikowska Ż. Diversity and multi-target potential of pyrazole, imidazole or triazole derivatives in modern anticancer therapy. Int J Mol Sci. 2026;27(10):4172. 10.3390/ijms27104172 PMC13206918 42196157

[59] Ajmal M, Mahato AK, Khan M, Rawat S, Husain A, Almalki EB, Alzahrani MA, Haque A, Hakme MJM, Albalawi AS, et al. Significance of triazole in medicinal chemistry: advancement in drug design, reward and biological activity. Chem Biodivers. 2024;21(7):e202400637. 10.1002/cbdv.202400637 38740555

[60] Isern JA, Carlucci R, Labadie GR, Porta EOJ. Progress and prospects of triazoles in advanced therapies for parasitic diseases. Trop Med Infect Dis. 2025;10(5):142. 10.3390/tropicalmed10050142 PMC12116058 40423371

[61] Kharb R, Haider K, Neha K, Yar MS. Aromatase inhibitors: role in postmenopausal breast cancer. Arch Pharm (Weinheim). 2020;353(8):e2000081. 10.1002/ardp.202000081 32449548

[62] Rashdan HRM, Shehadi IA. Triazoles synthesis & applications as nonsteroidal aromatase inhibitors for hormone-dependent breast cancer treatment. Heteroat Chem. 2022;2022:1–16.

[63] Nilkhet S, Vongthip W, Lertpatipanpong P, Prasansuklab A, Tencomnao T, Chuchawankul S, Baek SJ. Ergosterol inhibits the proliferation of breast cancer cells by suppressing AKT/GSK-3beta/beta-catenin pathway. Sci Rep. 2024;14(1):19664. 10.1038/s41598-024-70516-1 PMC11344128 39179606

[64] Lal K, Yadav P. Recent advancements in 1, 4-disubstituted 1H-1, 2, 3-triazoles as potential anticancer agents. Anticancer Agents Med Chem. 2018;18(1):21–37. 10.2174/1871520616666160811113531 27528183

[65] Odlo K, Fournier-Dit-Chabert J, Ducki S, Gani OABSM, Sylte I, Hansen TV. 1, 2, 3-Triazole analogs of combretastatin A-4 as potential microtubule-binding agents. Bioorg Med Chem. 2010;18(18):6874–6885. 10.1016/j.bmc.2010.07.032 20708408

[66] Maghraby MT-E, Mazyad Almutairi T, Bräse S, Salem OIA, Youssif BGM, Sheha MM. New 1, 2, 3-triazole/1, 2, 4-triazole hybrids as aromatase inhibitors: design, synthesis, and apoptotic antiproliferative activity. Molecules. 2023;28(20):7092. 10.3390/molecules28207092 PMC10609154 37894571

[67] Mokoena TP. Design and synthesis of quinoline-triazole schiff base metal complexes as potential anti-cancer agents [PhD Thesis]. 2023. [accessed 2026 Mar12]. http://ul.netd.ac.za/handle/10386/4611.

[68] Bhattacharya S, Adon T, Dsouza K, Kumar HY. Exploring the future of metal-based anticancer agents: a comprehensive review of ruthenium-based complexes. ChemistrySelect. 2025;10(9):e202404147.

[69] Priya S, Islam MM, Kasana S, Kurmi BD, Gupta GD, Patel P. Therapeutic potential of chalcone-1,2,3-triazole hybrids as anti-tumour agents: a systematic review and SAR studies. Future Med Chem. 2025;17(4):449–465. 10.1080/17568919.2025.2458450 PMC11834451 39886772

[70] Bagnolini G. Design and synthesis of RAD51-BRCA2 disruptors that trigger synthetic lethality in pancreatic cancer in combination with Olaparib. 2021. [accessed 2026 Apr 10]. https://amsdottorato.unibo.it/id/eprint/9589/.

[71] Matin MM, Matin P, Rahman MR, Ben Hadda T, Almalki FA, Mahmud S, Ghoneim MM, Alruwaily M, Alshehri S. Triazoles and their derivatives: chemistry, synthesis, and therapeutic applications. Front Mol Biosci. 2022;9:864286. 10.3389/fmolb.2022.864286 PMC9081720 35547394

[72] De Nino A, Maiuolo L, Costanzo P, Algieri V, Jiritano A, Olivito F, Tallarida MA. Recent progress in catalytic synthesis of 1, 2, 3-triazoles. Catalysts. 2021;11(9):1120.

[73] Vala DP, Vala RM, Patel HM. Versatile synthetic platform for 1,2,3-triazole chemistry. ACS Omega. 2022;7(42):36945–36987. 10.1021/acsomega.2c04883 PMC9608397 36312377

[74] Kaur R, Dwivedi AR, Kumar B, Kumar V. Recent developments on 1, 2, 4-triazole nucleus in anticancer compounds: a review. Anticancer Agents Med Chem. 2016;16(4):465–489. 10.2174/1871520615666150819121106 26286663

[75] González-Lainez M, Gallegos M, Munarriz J, Azpiroz R, Passarelli V, Jiménez MV, Pérez-Torrente JJ. Copper-catalyzed azide–alkyne cycloaddition (CuAAC) by functionalized NHC-based polynuclear catalysts: scope and mechanistic insights. Organometallics. 2022;41(15):2154–2169. 10.1021/acs.organomet.2c00246 PMC9374069 35971402

[76] Al-Salehi MA, Parmar MP, Rajani DP, Patel HM, Trivedi JH. Synthesis of 4-(3-(1-substituted/unsubstituted-phenyl-1H-1, 2, 3-triazole-4-yl) propoxy) benzaldehydes: in vitro antimicrobial, antimalarial activities and In silico docking study. J Mol Struct. 2024;1317:139006.

[77] Chi T-C, Yang P-C, Hung S-K, Wu H-W, Wang H-C, Liu H-K, Liu L-W, Chou H-H. Synthesis of multisubstituted 1,2,3-triazoles: regioselective formation and reaction mechanism. J Org Chem. 2024;89(8):5401–5408. 10.1021/acs.joc.3c02836 PMC11040735 38546539

[78] Couto Rodrigues S, Silva Moratório de Moraes R, Tavares de Almeida Pinto G, Miranda Martins MT, Antunes do Nascimento P, Alves Soares DL, Mestre Botelho AB, Cardoso Cruz C, Cunha AC. A review on chemistry and methods of synthesis of 1,2,4-triazole derivatives. Chem Rec. 2025;25(1):e202400190. 10.1002/tcr.202400190 39543436

[79] Dai J, Tian S, Yang X, Liu Z. Synthesis methods of 1, 2, 3-/1, 2, 4-triazoles: a review. Front Chem. 2022;10:891484. 10.3389/fchem.2022.891484 PMC9548580 36226121

[80] Swami S, Sharma N, Sharma G, Shrivastava R. Recent advances in microwave-assisted synthesis of triazoles and their derivatives: a green approach toward sustainable development methods. RSC Adv. 2025;15(4):2361–2415. 10.1039/d4ra06886f PMC11758809 39867335

[81] Thomas J, Dehaen W, John J. Advances in the synthesis of fused 1, 2, 3-triazoles via a MCR-intramolecular azide-alkyne cycloaddition Approach. Molecules. 2023;28:308. 10.3390/molecules28010308 PMC9821980 36615500

[82] Kloss F, Köhn U, Jahn BO, et al. Metal-Free 1,5-regioselective azide–alkyne [3 + 2]-cycloaddition. Chem – Asian J. 2011;6:2816–2824. 10.1002/asia.201100404 21882350

[83] Malik MS, Ahmed SA, Althagafi II, Ansari MA, Kamal A. Application of triazoles as bioisosteres and linkers in the development of microtubule targeting agents. RSC Med Chem. 2020;11(3):327–348. 10.1039/c9md00458k PMC7580775 33479639

[84] Rahman SMA, Bhatti JS, Thareja S, et al. Current development of 1, 2, 3-triazole derived potential antimalarial scaffolds: structure-activity relationship (SAR) and bioactive compounds. Eur J Med Chem. 2023;259:115699. 10.1016/j.ejmech.2023.115699 37542987

[85] Nguyen YT, Kim N, Lee HJ. Regulating histone deacetylases and carbonic anhydrases by metal complexes: a potent strategy for treating cancers. Discov Med. 2024;36(190):2152–2174. 10.24976/Discov.Med.202436190.198 39600271

[86] Rosam K, Monk BC, Lackner M. Sterol 14α-demethylase ligand-binding pocket-mediated acquired and intrinsic azole resistance in fungal pathogens. JoF. 2020;7(1):1. 10.3390/jof7010001 PMC7822023 33374996

[87] Aggarwal R, Sumran G. An insight on medicinal attributes of 1, 2, 4-triazoles. Eur J Med Chem. 2020;205:112652. 10.1016/j.ejmech.2020.112652 PMC7384432 32771798

[88] Rathod B, Kumar K. Synthetic and medicinal perspective of 1,2,4-triazole as anticancer agents. Chem Biodivers. 2022;19(11):e202200679. 10.1002/cbdv.202200679 36226542

[89] Akhter N, Batool S, Khan SG, Rasool N, Anjum F, Rasul A, Adem Ş, Mahmood S, Rehman A u, Nisa M u, et al. Bio-oriented synthesis and molecular docking studies of 1, 2, 4-triazole based derivatives as potential anti-cancer agents against HepG2 cell line. Pharmaceuticals. 2023;16(2):211. 10.3390/ph16020211 PMC9964635 37259360

[90] Acheampong F, Ostlund T, Hedge E, Laddusaw J, Alotaibi F, Elshaier YAMM, Halaweish F. Triazole-estradiol analogs induce apoptosis and inhibit EGFR and its downstream pathways in triple negative breast cancer. Molecules. 2025;30(3):605. 10.3390/molecules30030605 PMC11820259 39942711

[91] Ahmed SA, Mendonca P, Messeha SS, Soliman KFA. Anticancer effects of fucoxanthin through cell cycle arrest, apoptosis induction, and angiogenesis inhibition in triple-negative breast cancer cells. Molecules. 2023;28(18):6536. 10.3390/molecules28186536 PMC10535858 37764312

[92] Das A, Narayanam MK, Paul S, Mukhnerjee P, Ghosh S, Dastidar DG, Chakrabarty S, Ganguli A, Basu B, Pal M, et al. A novel triazole, NMK-T-057, induces autophagic cell death in breast cancer cells by inhibiting γ-secretase–mediated activation of Notch signaling. J Biol Chem. 2019;294(17):6733–6750. 10.1074/jbc.RA119.007671 PMC6497959 30824542

[93] An Q, Huang L, Wang C, Wang D, Tu Y. New strategies to enhance the efficiency and precision of drug discovery. Front Pharmacol. 2025;16:1550158. 10.3389/fphar.2025.1550158 PMC11850385 40008135

[94] Chathangad SN, Vijayan VN, George JA, Sadhukhan S. Mitigating antimicrobial resistance through strategic design of multimodal antibacterial agents based on 1,2,3-triazole with click chemistry. ACS Bio Med Chem Au. 2025;5(4):acsbiomedchemau.4c00132–504. 10.1021/acsbiomedchemau.4c00132 PMC12371489 40860028

[95] Sebastian A, Pandey V, Mohan CD, Chia YT, Rangappa S, Mathai J, Baburajeev CP, Paricharak S, Mervin LH, Bulusu KC, et al. Novel adamantanyl-based thiadiazolyl pyrazoles targeting EGFR in triple-negative breast cancer. ACS Omega. 2016;1(6):1412–1424. 10.1021/acsomega.6b00251 PMC6044684 30023509

[96] Zhang H-P, Jiang R-Y, Zhu J-Y, Sun K-N, Huang Y, Zhou H-H, Zheng Y-B, Wang X-J. PI3K/AKT/mTOR signaling pathway: an important driver and therapeutic target in triple-negative breast cancer. Breast Cancer. 2024;31(4):539–551. 10.1007/s12282-024-01567-5 PMC11194209 38630392

[97] Topacio BR, Zatulovskiy E, Cristea S, Xie S, Tambo CS, Rubin SM, Sage J, Kõivomägi M, Skotheim JM. Cyclin D-Cdk4, 6 drives cell-cycle progression via the retinoblastoma protein’s C-terminal helix. Mol Cell. 2019;74(4):758–770.e4. 10.1016/j.molcel.2019.03.020 PMC6800134 30982746

[98] Chen B-J, Wu Y-L, Tanaka Y, Zhang W. Small molecules targeting c-Myc oncogene: promising anti-cancer therapeutics. Int J Biol Sci. 2014;10(10):1084–1096. 10.7150/ijbs.10190 PMC4202025 25332683

[99] Shawgo ME, Shelton SN, Robertson JD. Caspase-9 activation by the apoptosome is not required for fas-mediated apoptosis in type II Jurkat cells. J Biol Chem. 2009;284(48):33447–33455. 10.1074/jbc.M109.032359 PMC2785189 19758996

[100] Qian S, Wei Z, Yang W, Huang J, Yang Y, Wang J. The role of BCL-2 family proteins in regulating apoptosis and cancer therapy. Front Oncol. 2022;12:985363. 10.3389/fonc.2022.985363 PMC9597512 36313628

[101] Parrish AB, Freel CD, Kornbluth S. Cellular mechanisms controlling caspase activation and function. Cold Spring Harb Perspect Biol. 2013;5(6):a008672. 10.1101/cshperspect.a008672 PMC3660825 23732469

[102] Shama AR, Savaliya ML. Benzothiazole-triazole hybrids: novel anticancer agents inducing cell cycle arrest and apoptosis through Bcl-2 inhibition in triple-negative breast cancer. Bioorg Chem. 2025;155:108150. 10.1016/j.bioorg.2025.108150 39799730

[103] Duffy AM, Bouchier-Hayes DJ, Harmey JH. Vascular endothelial growth factor (VEGF) and its role in non-endothelial cells: autocrine signalling by VEGF. In Madame curie bioscience database [Internet]. Austin (TX): Landes Bioscience; 2013 [accessed 2025 May 29]. https://www.ncbi.nlm.nih.gov/books/NBK6482/.

[104] Rotenberg N, Feldman M, Shirian J, Hockla A, Radisky ES, Shifman JM. Engineered TIMP2 with narrow MMP-9 specificity is an effective inhibitor of invasion and proliferation of triple-negative breast cancer cells. J Biol Chem. 2024;300(11):107867. 10.1016/j.jbc.2024.107867 PMC11609464 39419285

[105] Ang HL, Mohan CD, Shanmugam MK, Leong HC, Makvandi P, Rangappa KS, Bishayee A, Kumar AP, Sethi G. Mechanism of epithelial-mesenchymal transition in cancer and its regulation by natural compounds. Med Res Rev. 2023;43(4):1141–1200. 10.1002/med.21948 36929669

[106] Ana G, Kelly PM, Malebari AM, Noorani S, Nathwani SM, Twamley B, Fayne D, O’Boyle NM, Zisterer DM, Pimentel EF, et al. Synthesis and biological evaluation of 1-(diarylmethyl)-1 H-1, 2, 4-triazoles and 1-(diarylmethyl)-1 H-imidazoles as a novel class of anti-mitotic agent for activity in breast cancer. Pharmaceuticals. 2021;14(2):169. 10.3390/ph14020169 PMC7926793 33671674

[107] Deb M, Singh H, Manhas D, Nandi U, Guru SK, Das P. Development of di-arylated 1, 2, 4-triazole-based derivatives as therapeutic agents against breast cancer: synthesis and biological evaluation. RSC Med Chem. 2024;15(9):3097–3113. 10.1039/d4md00285g PMC11411613 39309354

[108] Abd El Salam HA, Abu-Shahba N, Ibrahim Fouad G, Mahmoud M, Mostafa EA, Abozeid MAM, Abo-Salem HM, Azouz RAM. New indole-linked 1, 2, 4-triazole derivatives as dual FAK inhibitors and apoptosis inducers targeting survival and migration in triple-negative breast cancer in-vitro. Sci Rep. 2026;16(1):13134. 10.1038/s41598-026-41032-1 PMC13103447 42020433

[109] Elrashedy A, Ibrahim NE, Abo-Salem H, Elaasser MM, El-Sawy ER. Design, synthesis, and molecular modeling of new 1, 2, 4-triazole-containing indole compounds as aromatase antagonists for the treatment of breast cancer. Bioorg Chem. 2025;163:108677. 10.1016/j.bioorg.2025.108677 40489920

[110] Yang J, Nie J, Ma X, Wei Y, Peng Y, Wei X. Targeting PI3K in cancer: mechanisms and advances in clinical trials. Mol Cancer. 2019;18(1):26. 10.1186/s12943-019-0954-x PMC6379961 30782187

[111] Lawrence T. The nuclear factor NF-κB pathway in inflammation. Cold Spring Harb Perspect Biol. 2009;1(6):a001651–a001651. 10.1101/cshperspect.a001651 PMC2882124 20457564

[112] Yue J, López JM. Understanding MAPK signaling pathways in apoptosis. Int J Mol Sci. 2020;21(7):2346. 10.3390/ijms21072346 PMC7177758 32231094

[113] Draoui Y, Radi S, Bahjou Y, Idir A, El Mahdaoui A, Zyad A, Miras HN, Ferbinteanu M, Rotaru A, Garcia Y, et al. New triazole-based coordination complexes as antitumor agents against triple negative breast cancer MDA-MB-468 cell line. RSC Adv. 2023;13(51):36158–36167. 10.1039/d3ra07714d PMC10714199 38090097

[114] Hamdy R, Jones AT, El-Sadek M, Hamoda AM, Shakartalla SB, Al Shareef ZM, Soliman SSM, Westwell AD. New bioactive fused triazolothiadiazoles as Bcl-2-targeted anticancer agents. Int J Mol Sci. 2021;22(22):12272. 10.3390/ijms222212272 PMC8621373 34830153

[115] Pereira D, Pinto M, Correia-da-Silva M, Cidade H. Recent advances in bioactive flavonoid hybrids linked by 1, 2, 3-triazole ring obtained by click chemistry. Molecules. 2021;27(1):230. 10.3390/molecules27010230 PMC8746422 35011463

[116] Montero V, Montana M, Carré M, Vanelle P. Quinoxaline derivatives: recent discoveries and development strategies towards anticancer agents. Eur J Med Chem. 2024;271:116360. 10.1016/j.ejmech.2024.116360 38614060

[117] Bass AKA, El-Zoghbi MS, Nageeb E-SM, Mohamed MFA, Badr M, Abuo-Rahma GE-DA. Comprehensive review for anticancer hybridized multitargeting HDAC inhibitors. Eur J Med Chem. 2021;209:112904. 10.1016/j.ejmech.2020.112904 33077264

[118] Augsten LV, Göethel G, Gauer B, Feiffer Charão M, von Poser G, Canto RFS, Arbo MD, Eifler-Lima VL, Garcia SC. Antiproliferative activity and toxicity evaluation of 1, 2, 3-triazole and 4-methyl coumarin hybrids in the MCF7 breast cancer cell line. RSC Med Chem. 2023;14(5):869–879. 10.1039/d3md00031a PMC10211326 37252094

[119] Guo H-Y, Chen Z-A, Shen Q-K, Quan Z-S. Application of triazoles in the structural modification of natural products. J Enzyme Inhib Med Chem. 2021;36(1):1115–1144. 10.1080/14756366.2021.1890066 PMC8231395 34167422

[120] Leśniewska A, Przybylski P. Seven-membered N-heterocycles as approved drugs and promising leads in medicinal chemistry as well as the metal-free domino access to their scaffolds. Eur J Med Chem. 2024;275:116556. 10.1016/j.ejmech.2024.116556 38879971

[121] Pastuch-Gawołek G, Szreder J, Domińska M, Pielok M, Cichy P, Grymel M. A small sugar molecule with huge potential in targeted cancer therapy. Pharmaceutics. 2023;15(3):913. 10.3390/pharmaceutics15030913 PMC10056414 36986774

[122] Hamad HT. The anti-cancer effectiveness of some heterocyclic compounds containing sulfur atom. Results Chem. 2025;15:102182.

[123] Temml V, Kutil Z. Structure-based molecular modeling in SAR analysis and lead optimization. Comput Struct Biotechnol J. 2021;19:1431–1444. 10.1016/j.csbj.2021.02.018 PMC7979990 33777339

[124] Rahman R, Fonseka AD, Sua S-C, Ahmad M, Rajendran R, Ambu S, Davamani F, Khoo AS-B, Chitra E. Inhibition of breast cancer xenografts in a mouse model and the induction of apoptosis in multiple breast cancer cell lines by lactoferricin B peptide. J Cell Mol Med. 2021;25(15):7181–7189. 10.1111/jcmm.16748 PMC8335703 34236134

[125] Ocal I, Avci A, Cakalagaoglu F, Can H. Lack of correlations among histopathological parameters, Ki-67 proliferation index and prognosis in pheochromocytoma patients. Asian Pac J Cancer Prev. 2014;15(4):1751–1755. 10.7314/apjcp.2014.15.4.1751 24641403

[126] Peng Z, Lv X, Sun H, Zhao L, Huang S. 3D tumor cultures for drug resistance and screening development in clinical applications. Mol Cancer. 2025;24(1):93. 10.1186/s12943-025-02281-2 PMC11927140 40119343

[127] Akash S, Aovi FI, Azad MAK, Kumer A, Chakma U, Islam MR, Mukerjee N, Rahman MM, Bayıl I, Rashid S, et al. A drug design strategy based on molecular docking and molecular dynamics simulations applied to development of inhibitor against triple-negative breast cancer by scutellarein derivatives. PLoS One. 2023;18(10):e0283271. 10.1371/journal.pone.0283271 PMC10569544 37824496

[128] Trott O, Olson AJ. AutoDock Vina: improving the speed and accuracy of docking with a new scoring function, efficient optimization, and multithreading. J Comput Chem. 2010;31(2):455–461. 10.1002/jcc.21334 PMC3041641 19499576

[129] Verdonk ML, Cole JC, Hartshorn MJ, Murray CW, Taylor RD. Improved protein–ligand docking using GOLD. Proteins Struct Funct Bioinforma. 2003;52(4):609–623. 10.1002/prot.10465 12910460

[130] Almutairi HH, Alharbi A, Alanzy AL, Alomari K, Aljowni MA, Alessa A, Katouah HA, El-Metwaly N. Synthesis and molecular modeling of new triazole-oxadiazole and triazole-thiadiazole compounds as cytotoxic agents. Arab J Chem. 2026;19:7062025.

[131] Das A, Prajapati A, Karna A, Sharma HK, Uppal S, Lather V, Pandita D, Agarwal P. Structure-based virtual screening of chemical libraries as potential MELK inhibitors and their therapeutic evaluation against breast cancer. Chem Biol Interact. 2023;376:110443. 10.1016/j.cbi.2023.110443 36893906

[132] Ibezim A, Madukaife MS, Osigwe SC, Engel N, Karuppasamy R, Ntie-Kang F. Fragment-based virtual screening discovers potential new Plasmodium PI4KIIIβ ligands. BMC Chem. 2022;16(1):19. 10.1186/s13065-022-00812-2 PMC8944149 35331319

[133] Opo FADM, Moulay M, Zari A, Alqaderi A, Alkarim S, Zari T, Bhuiyan MA, Mahmoud MM, Aljoud F, Suhail M, et al. Pharmacophore-based virtual screening approaches to identify novel molecular candidates against EGFR through comprehensive computational approaches and in-vitro studies. Front Pharmacol. 2022;13:1027890. 10.3389/fphar.2022.1027890 PMC9707641 36457709

[134] Lombardo F, Desai PV, Arimoto R, Desino KE, Fischer H, Keefer CE, Petersson C, Winiwarter S, Broccatelli F. *In Silico* absorption, distribution, metabolism, excretion, and pharmacokinetics (ADME-PK): utility and best practices. An industry perspective from the international consortium for innovation through quality in pharmaceutical development: miniperspective. J Med Chem. 2017;60(22):9097–9113. 10.1021/acs.jmedchem.7b00487 28609624

[135] Doak BC, Over B, Giordanetto F, Kihlberg J. Oral druggable space beyond the rule of 5: insights from drugs and clinical candidates. Chem Biol. 2014;21(9):1115–1142. 10.1016/j.chembiol.2014.08.013 25237858

[136] Bhat V, Chatterjee J. The use of *In Silico* tools for the toxicity prediction of potential inhibitors of SARS-CoV-2. Altern Lab Anim. 2021;49(1-2):22–32. 10.1177/02611929211008196 PMC8047515 33845649

[137] Sun L, Yang H, Li J, Wang T, Li W, Liu G, Tang Y. In silico prediction of compounds binding to human plasma proteins by QSAR models. ChemMedChem. 2018;13(6):572–581. 10.1002/cmdc.201700582 29057587

[138] Batool Z, Kamal MA, Shen B. Advancements in triple-negative breast cancer sub-typing, diagnosis and treatment with assistance of artificial intelligence: a focused review. J Cancer Res Clin Oncol. 2024;150(8):383. 10.1007/s00432-024-05903-2 PMC11300496 39103624

[139] Gao P, Zhang J, Sun Y, Yu J. Accurate predictions of aqueous solubility of drug molecules via the multilevel graph convolutional network (MGCN) and SchNet architectures. Phys Chem Chem Phys. 2020;22(41):23766–23772. 10.1039/d0cp03596c 33063077

[140] Sousa T, Correia J, Pereira V, Rocha M. Generative deep learning for targeted compound design. J Chem Inf Model. 2021;61(11):5343–5361. 10.1021/acs.jcim.0c01496 34699719

[141] Swanson K, Walther P, Leitz J, Mukherjee S, Wu JC, Shivnaraine RV, Zou J. ADMET-AI: a machine learning ADMET platform for evaluation of large-scale chemical libraries. Bioinformatics. 2024;40(7):btae416. 10.1093/bioinformatics/btae416 PMC11226862 38913862

[142] Xu G-Q, Gong X-Q, Zhu Y-Y, Yao X-J, Peng L-Z, Sun G, Yang J-X, Mao L-F. Novel 1, 2, 3-triazole erlotinib derivatives as potent IDO1 inhibitors: design, drug-target interactions prediction, synthesis, biological evaluation, molecular docking and ADME properties studies. Front Pharmacol. 2022;13:854965. 10.3389/fphar.2022.854965 PMC9168369 35677437

[143] Maccallini C, Gallorini M, Sisto F, Akdemir A, Ammazzalorso A, De Filippis B, Fantacuzzi M, Giampietro L, Carradori S, Cataldi A, et al. New azolyl-derivatives as multitargeting agents against breast cancer and fungal infections: synthesis, biological evaluation and docking study. J Enzyme Inhib Med Chem. 2021;36(1):1632–1645. 10.1080/14756366.2021.1954918 PMC8300937 34289751

[144] Simiczyjew A, Dratkiewicz E, Van Troys M, Ampe C, Styczeń I, Nowak D. Combination of EGFR inhibitor lapatinib and MET inhibitor foretinib inhibits migration of triple negative breast cancer cell lines. Cancers (Basel). 2018;10(9):335. 10.3390/cancers10090335 PMC6162814 30227653

[145] Vinod BS, Antony J, Nair HH, Puliyappadamba VT, Saikia M, Narayanan SS, Bevin A, Anto RJ. Mechanistic evaluation of the signaling events regulating curcumin-mediated chemosensitization of breast cancer cells to 5-fluorouracil. Cell Death Dis. 2013;4(2):e505. e505. 10.1038/cddis.2013.26 PMC3734809 23429291

[146] Saddam M, Paul SK, Habib MA, Fahim MA, Mimi A, Islam S, Paul B, Helal MMU. Emerging biomarkers and potential therapeutics of the BCL-2 protein family: the apoptotic and anti-apoptotic context. Egypt J Med Hum Genet. 2024;25(1):12.

[147] Saranya T, Kavithaa K, Paulpandi M, Ramya S, Preethi S, Balachandar V, Narayanasamy A. Enhanced apoptogenesis and oncogene regulatory mechanism of troxerutin in triple negative breast cancer cells. Toxicol Res (Camb). 2020;9(3):230–238. 10.1093/toxres/tfaa029 PMC7329179 32670554

[148] Huang A, Xue H, Xie T, Xiang L, Chen Z, Ma A, Yan H, Yuan J. A review of the pathogenesis of mitochondria in breast cancer and progress of targeting mitochondria for breast cancer treatment. J Transl Med. 2025;23(1):70. 10.1186/s12967-025-06077-2 PMC11734335 39815317

[149] Khan A, Naaz F, Basit R, Das D, Bisht P, Shaikh M, Lone BA, Pokharel YR, Ahmed QN, Parveen S, et al. 1,2,3-triazole tethered hybrid capsaicinoids as antiproliferative agents active against lung cancer cells (A549). ACS Omega. 2022;7(36):32078–32100. 10.1021/acsomega.2c03325 PMC9476207 36119972

[150] Diana A, Carlino F, Franzese E, Oikonomidou O, Criscitiello C, De Vita F, Ciardiello F, Orditura M. Early triple negative breast cancer: conventional treatment and emerging therapeutic landscapes. Cancers (Basel). 2020;12(4):819. 10.3390/cancers12040819 PMC7225917 32235297

[151] Avula SK, Rehman NU, Khan F, Ullah O, Halim SA, Khan A, Anwar MU, Rahman SM, Csuk R, Al-Harrasi A, et al. Triazole-tethered boswellic acid derivatives against breast cancer: synthesis, in vitro, and in-silico studies. J Mol Struct. 2023;1282:135181.

[152] Kinnel B, Singh SK, Oprea-Ilies G, Singh R. Targeted therapy and mechanisms of drug resistance in breast cancer. Cancers (Basel). 2023;15(4):1320. 10.3390/cancers15041320 PMC9954028 36831661

[153] Alam MM. 1, 2, 3-Triazole hybrids as anticancer agents: a review. Arch Pharm (Weinheim). 2022;355(1):e2100158. 10.1002/ardp.202100158 34559414

[154] Choi Y, Yu A-M. ABC transporters in multidrug resistance and pharmacokinetics, and strategies for drug development. Curr Pharm Des. 2014;20(5):793–807. 10.2174/138161282005140214165212 PMC6341993 23688078

[155] Wang S-Q, Teng Q-X, Wang S, Lei Z-N, Hu H-H, Lv H-F, Chen B-B, Wang J-Z, Shi X-J, Xu W-F, et al. Preclinical studies of the triazolo [1, 5-a] pyrimidine derivative WS-716 as a highly potent, specific and orally active P-glycoprotein (P-gp) inhibitor. Acta Pharm Sin B. 2022;12(8):3263–3280. 10.1016/j.apsb.2022.03.023 PMC9366537 35967279

[156] Pires MM, Emmert D, Hrycyna CA, Chmielewski J. Inhibition of P-glycoprotein-mediated paclitaxel resistance by reversibly linked quinine homodimers. Mol Pharmacol. 2009;75(1):92–100. 10.1124/mol.108.050492 PMC2685053 18945821

[157] Ahmad I. Therapeutic significance of molecular hybrids for breast cancer research and treatment. RSC Med Chem. 2023;14:218–238. 10.1039/d2md00356b PMC9945856 36846377

[158] Takebe N, Miele L, Harris PJ, Jeong W, Bando H, Kahn M, Yang SX, Ivy SP. Targeting Notch, Hedgehog, and Wnt pathways in cancer stem cells: clinical update. Nat Rev Clin Oncol. 2015;12(8):445–464. 10.1038/nrclinonc.2015.61 PMC4520755 25850553

[159] Sun N, Yang K, Yan W, Yao M, Yu C, Duan W, Gu X, Guo D, Jiang H, Xie C, et al. Design and synthesis of triazole-containing HDAC inhibitors that induce antitumor effects and immune response. J Med Chem. 2023;66(7):4802–4826. 10.1021/acs.jmedchem.2c01985 36934335

[160] Asleh K, Riaz N, Nielsen TO. Heterogeneity of triple negative breast cancer: current advances in subtyping and treatment implications. J Exp Clin Cancer Res. 2022;41(1):265. 10.1186/s13046-022-02476-1 PMC9434975 36050786

[161] Lengerli D, Ibis K, Nural Y, Banoglu E. The 1,2,3-triazole ‘all-in-one’ ring system in drug discovery: a good bioisostere, a good pharmacophore, a good linker, and a versatile synthetic tool. Expert Opin Drug Discov. 2022;17(11):1209–1236. 10.1080/17460441.2022.2129613 36164263

[162] Wang K, Huang Y, Wang Y, You Q, Wang L. Recent advances from computer-aided drug design to artificial intelligence drug design. RSC Med Chem. 2024;15(12):3978–4000. 10.1039/d4md00522h PMC11523840 39493228

[163] Anwar DM, Hedeya HY, Ghozlan SH, Ewas BM, Khattab SN. Surface-modified lipid-based nanocarriers as a pivotal delivery approach for cancer therapy: application and recent advances in targeted cancer treatment. Beni-Suef Univ J Basic Appl Sci. 2024;13(1):106.

[164] Ansari AM, Sharma NK, Tripathi K, Bhardwaj G, Chauhan R, Tonk RK, Shahwan M, Tuli HS, Chauhan A. Overcoming multifaceted challenges in cancer treatment: targeting signal transduction pathways and tumor microenvironment for enhanced therapeutic efficacy. J Appl Pharm Sci. 2025;15:040–063.

[165] Wu Y-J, Meanwell NA. Geminal diheteroatomic motifs: some applications of acetals, ketals, and their sulfur and nitrogen homologues in medicinal chemistry and drug design. J Med Chem. 2021;64(14):9786–9874. 10.1021/acs.jmedchem.1c00790 34213340

[166] Czyrski A, Resztak M, Świderski P, Brylak J, Główka FK. The overview on the pharmacokinetic and pharmacodynamic interactions of triazoles. Pharmaceutics. 2021;13(11):1961. 10.3390/pharmaceutics13111961 PMC8620887 34834376

[167] Salata GC, Hirokawa CM, de Melo GB, Malagó ID, Nunes JR, Passos JS, Daré RG, Lopes LB. In vitro methodologies to evaluate nanocarriers for cancer treatment: where are we? Cancer Nano. 2025;16(1):21.

[168] Khan J, Rani A, Aslam M, Maharia RS, Pandey G, Nand B. Exploring triazole-based drugs: synthesis, application, FDA approvals, and clinical trial updates–A comprehensive review. Tetrahedron. 2024;162:134122.

[169] Yu M, Lu X. Dual-target therapeutic strategies in triple-negative breast cancer: mechanistic insights and clinical potential. Cancers (Basel). 2025;17(21):3455. 10.3390/cancers17213455 PMC12607794 41228247

